# Shared and distinct metabolomics profiles associated with microvascular complications in the Diabetes Prevention Program Outcomes Study

**DOI:** 10.1007/s00125-025-06571-8

**Published:** 2025-10-11

**Authors:** Wei Perng, Shiyu Shu, David M. Nathan, Jose A. Luchsinger, Robert E. Gerszten, Roeland J. W. Middelbeek, Steven E. Kahn, William C. Knowler, Dana Dabelea, Marinella Temprosa

**Affiliations:** 1 Lifecourse Epidemiology of Adiposity and Diabetes (LEAD) Center, Department of Epidemiology at the Colorado School of Public Health and the University of Colorado Anschutz Medical Campus, Aurora, CO USA; 2 Department of Biostatistics and Bioinformatics, Milken Institute School of Public Health, George Washington University, Rockville, MD USA; 3 Diabetes Center, Massachusetts General Hospital and Harvard Medical School, Boston, MA USA; 4 Departments of Medicine and Epidemiology, Columbia University Irving Medical Center, New York, NY USA; 5 Beth Israel Deaconess Medical Center, Harvard Medical School, Boston, MA USA; 6 Joslin Diabetes Center, Harvard Medical School, Boston, MA USA; 7 Division of Metabolism, Endocrinology and Nutrition, VA Puget Sound Health Care System and University of Washington, Seattle, WA USA; 8 Consultant, Department of Biostatistics and Bioinformatics, Milken Institute School of Public Health, George Washington University, Rockville, MD USA; 9 Department of Pediatrics, School of Medicine, University of Colorado Anschutz Medical Campus, Aurora, CO USA

**Keywords:** Cohort study, Metabolomics, Microvascular complications, Neuropathy, Retinopathy

## Abstract

**Aims/hypothesis:**

The aim of this study was to identify shared and distinct metabolite profiles prospectively associated with nephropathy, retinopathy and neuropathy at 15 years’ follow-up among 1947 participants in the Diabetes Prevention Program Outcomes Study, the long-term follow-up of the Diabetes Prevention Program (DPP).

**Methods:**

We applied bootstrapped LASSO to 353 annotated metabolites to identify metabolites associated with one or more complication. For these metabolite hits, we tested for an interaction with DPP treatment arm, and ran multivariable models for the pooled sample or within treatment group as appropriate.

**Results:**

At follow-up, 572 participants had one or more complication (*n*=277 nephropathy, *n*=194 retinopathy, *n*=212 neuropathy). Of 105 metabolites that predicted any complication, 74 predicted one, 27 predicted two, and four predicted all three. In a pooled analysis of 69 metabolites without treatment arm interactions, histidine predicted lower odds of nephropathy (OR 0.75; 95% CI 0.69, 0.88), and serine predicted lower odds of nephropathy (OR 0.69; 95% CI 0.58, 0.82) and neuropathy (OR 0.68; 95% CI 0.56, 0.84). Of 36 metabolites that interacted with treatment arm, higher *N*-carbamoyl-β-alanine predicted greater odds of nephropathy (OR 1.99; 95% CI 1.38, 2.99) and C22:0-sphingomyelin predicted lower odds of neuropathy (OR 0.54; 95% CI 0.37, 0.77) in the metformin arm. In the lifestyle intervention arm, quinolinic acid predicted greater odds of neuropathy (OR 1.64; 95% CI 1.24, 2.19). These estimates accounted for sex, race, baseline age, BMI and smoking, and time elapsed during follow-up. Further adjustment for HbA_1c_ during follow-up, incident diabetes and eGFR did not change the results.

**Conclusions/interpretation:**

The existence of distinct metabolite profiles associated with single microvascular complications highlights the importance of characterising pathophysiological mechanisms specific to each complication, in addition to studying shared mechanisms across multiple complications.

**Graphical Abstract:**

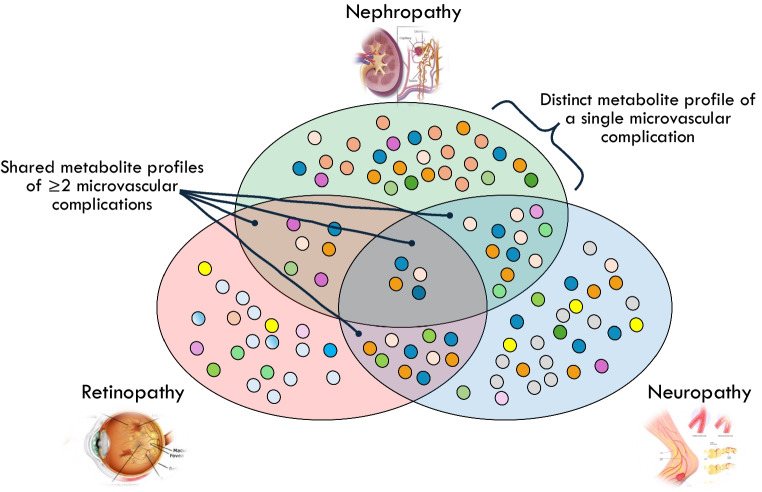

**Supplementary Information:**

The online version of this article (10.1007/s00125-025-06571-8) contains peer-reviewed but unedited supplementary material.


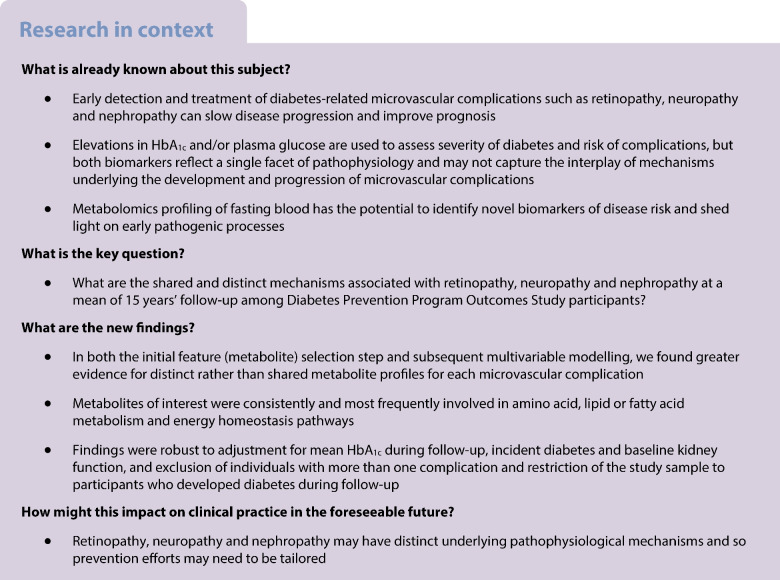


## Introduction

Diabetes affects one in ten individuals worldwide, with most individuals having type 2 diabetes [1]. Having diabetes can lead to debilitating long-term microvascular complications, including neuropathy, retinopathy and nephropathy. Early detection and treatment of such complications can slow disease progression and improve prognosis, making it crucial to identify biomarkers of risk early in pathogenesis [2].

At present, elevations in HbA_1c_ or plasma glucose are used to assess diabetes severity and the risk of complications in clinical settings [3]. However, both biomarkers reflect a single facet of pathophysiology that may not capture the interplay of mechanisms underlying the development and progression of microvascular complications. Further, microvascular complications may be present at the time of diabetes diagnosis [4]. This occurrence, which is thought to reflect the progression of hyperglycaemia or organ damage due to metabolic comorbidities but may also be a result of other mechanisms that operate independently of or jointly with hyperglycaemia (e.g. mitochondrial dysfunction, oxidative stress, inflammation and genetic predisposition), underscores the need to identify early biomarkers for risk of microvascular complications, not only among individuals with diabetes but also in those who do not yet have the disease.

Metabolomics, or the systematic study of low molecular weight compounds in biological tissues, is a promising tool for biomarker discovery. Analysing metabolites in blood is especially useful for identifying functional biomarkers of disease risk as these circulating profiles integrate an individual’s intrinsic physiology with external exposures and risk factors [5]. Accordingly, metabolite profiles not only provide information about disease risk, progression and prognosis, but also have potential to serve as therapeutic targets [6].

Here, we capitalise on data from the Diabetes Prevention Program Outcomes Study (DPPOS), a long-term follow-up of the Diabetes Prevention Program (DPP) [7], a randomised clinical trial of lifestyle intervention or metformin for the prevention of diabetes among adults at high risk for type 2 diabetes owing to the presence of prediabetes and overweight/obesity. Our objective was to identify shared and unique metabolite profiles that were detectable at baseline (i.e. the time of randomisation into treatment arms for the DPP) and were associated with prevalent nephropathy, neuropathy and retinopathy after a mean of 15 years’ follow-up. We hypothesised that we would identify metabolite profiles that are specific to single complications and also shared metabolite profiles across multiple complications.

## Methods

### Study population

The DPP was a randomised, clinical trial that ran from 1996–2001 at 27 US centres among participants at high risk of developing diabetes owing to overweight/obesity, impaired glucose tolerance and a fasting glucose of 5.3–6.9 mmol/l (except in the American Indian clinics) [8]. During the main phase of the trial, 3234 participants were randomised to intensive lifestyle intervention, metformin or placebo. The main findings of the DPP were that intensive lifestyle intervention and metformin reduced the incidence of diabetes by 58% and 31%, respectively, vs the placebo, after 2.8 years’ follow-up [7].

The current study included 2015 DPP participants enrolled in the DPPOS (ClinicalTrials.gov NCT00038727) who had metabolomics assays performed on baseline samples. Sample selection for metabolite profiling followed a nested case–control design, with 1:1 matching of participants with incident diabetes vs those without during the DPP for sex (a biological variable for which chronic disease progression and risk vary), original DPP treatment group, hypertension status, and a propensity score calculated as: logit (diabetes status) = age at randomisation + race/ethnicity + baseline fasting plasma glucose + BMI, using data available by 31 July 2001 [9]. The present study is a complete case analysis of 1947 participants for whom metabolomics data are available and for whom we have information on microvascular complications at DPPOS phase 2 (2008–2015), i.e. after a mean of 15 years’ follow-up [10]. The institutional review board at each clinical centre approved the protocol. All participants provided written informed consent.

### Metabolomics

The three platforms used for metabolomics profiling have been described previously [11, 12] and details are provided in the electronic supplementary material (ESM) Methods.

### Microvascular complications

The outcomes of interest were prevalent nephropathy, retinopathy and neuropathy assessed at a mean of 15 years after baseline [10]. Nephropathy was defined (1) using an albuminuria threshold of ≥30 mg albumin per g creatinine in a spot urine collection on two consecutive tests; (2) an eGFR <45 ml/min per 1.73 m^2^, based on annual calculation of serum creatinine levels using the CKD-EPI equation [13]; or (3) renal failure based on end-stage renal disease, dialysis or transplantation. Participants taking blood pressure-lowering medications at the final assessment who did not meet the albuminuria or eGFR criteria were considered to have nephropathy if the criteria were met at two consecutive past visits. Retinopathy was diagnosed using seven-field stereoscopic fundus photography if early treatment diabetic retinopathy study grade ≥20 [14] was present in either eye or if retinopathy had been treated with laser or intravitreal injections. Neuropathy was diagnosed based on loss of light touch sensation (fewer than eight of ten applications to the great toe dorsum) measured using a 10 g Semmes–Weinstein monofilament.

### Covariates

At baseline, an interviewer-administered questionnaire was used to assess smoking habits, age, sex and race/ethnicity [15]. Clinic staff measured weight in duplicate using a calibrated scale and standing height in duplicate using a standard stadiometer. These values were used to calculate BMI. Fasting blood was obtained at baseline and at each year of follow-up for HbA_1c_ assessment. During follow-up, a diabetes diagnosis was made if fasting plasma glucose was ≥7.0 mmol/l at the 6-monthly examination or based on a 2 h plasma glucose level ≥11.1 mmol/l during the annual 75 g OGTT, both requiring confirmation by a repeat test.

### Data analysis

**Fig. 1:**
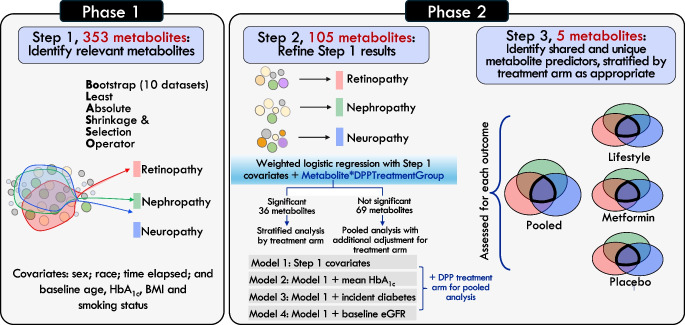
Overview of the two-phase analytical approach

We carried out the analysis in two phases (Fig. 1). The first phase was feature selection, in which we identified metabolites associated with each microvascular outcome using the least absolute shrinkage and selection operator (LASSO) [16]. LASSO is a regularised regression technique that is designed to identify the strongest predictor–outcome associations from a high-dimensional, correlated set of predictors (metabolites) via a tuning parameter λ. Estimates <|λ| are considered irrelevant, even if statistically significant, and are removed from the model to prevent type 1 error. We entered the 353 metabolites, standardised as *z* scores, as predictors in the LASSO. Each microvascular outcome was assessed separately as the dependent variable. To further reduce type 1 error, we created ten bootstrapped copies of the original metabolite dataset, implemented the LASSO model in each resampled dataset, and retained metabolites selected by the LASSO seven or more of the ten times for each outcome [17]. In all models, we accounted for race/ethnicity, time elapsed during follow-up, and baseline age, HbA_1c_, BMI and smoking status. After identifying metabolite hits, we examined the list of metabolites for each complication, and identified those that were specific to one outcome, shared across pairs of outcomes, or shared across all three outcomes, for subsequent analysis.

The second phase of the analysis considered the heterogeneity of effects in metabolite–outcome associations, and investigated the robustness of metabolite–outcome associations via multivariable adjustment. We first determined which metabolites could be modelled in pooled analysis vs those that required stratification by DPP treatment arm. The need for this step was suggested by a prior DPPOS study that found heterogeneity of effects in the association of metabolites with incident diabetes by treatment arm [18]. Here, we used a weighted logistic regression model that accounted for the inverse of each participant’s propensity score for having metabolomics data (and thus the propensity of being included in this analysis) to mitigate selection bias [18], a product term between each metabolite and an indicator for treatment arm, and the main effects for each. If the type 3 test for the product term was significant after correction for false discovery rate (FDR), then this suggested heterogeneity of effects of that metabolite by treatment arm and the need to run subsequent multivariable models within treatment arm.

For the multivariable models, model 1 included sex, race, time elapsed, and baseline age, HbA_1c_, BMI and smoking status, plus treatment arm for metabolites assessed via pooled analysis. Model 2 was the same as model 1 plus mean HbA_1c_ during follow-up. Model 3 was the same as model 1 plus diabetes status at the time of microvascular outcome assessment. Model 4 was the same as model 1 plus baseline eGFR. We focused on model 1 when further honing the metabolite list from those selected from phase 1 (105 metabolites), while applying FDR correction. Subsequently, we ran models 2–4 only for metabolites that met the FDR-corrected α threshold of 0.05 in model 1. Our rationale for focusing on model 1 was because covariates in later models could be mediators and thus introduce collider stratification bias into estimates.

We conducted two sensitivity analyses. First, we re-ran all models after excluding 107 participants with one or more complication at follow-up to rule out the possibility of metabolite profiles being shared among complications because they occurred in the same individual. Second, we re-ran all models only among the 952 individuals who developed diabetes to better ensure that metabolite–outcome associations reflect progression of hyperglycaemia. For both sensitivity analyses, we compared the distribution of hits, pathways and key metabolites to the main analysis.

We performed all analyses using R version 4.3.0 (R Foundation for Statistical Computing, Vienna, Austria).

## Results

**Fig. 2:**
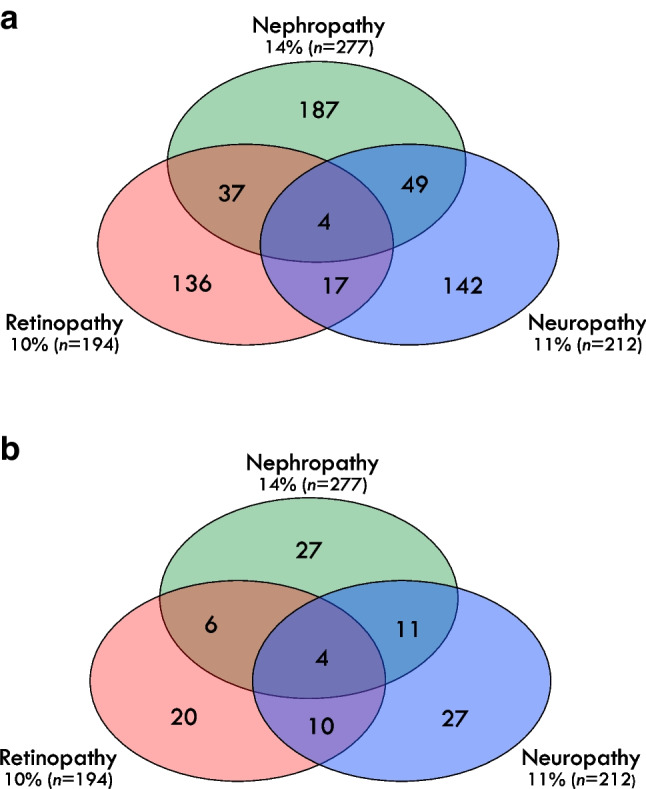
(**a**) Distribution of microvascular complications among 572 DPPOS participants who had at least one complication at a mean of 15 years of follow-up. (**b**) Distribution of 105 metabolites associated with microvascular complications

At the time of randomisation into DPP treatment arms (baseline), participants were 53.1 ± 10.1 years old. The majority were non-Hispanic White (57%; 1104/1947) and female (67%; 1298/1947). Additional characteristics are shown in ESM Table 1. After a mean of 15 years’ follow-up, 572 participants (29%) had one or more microvascular complication, including 277 with nephropathy, 194 with retinopathy and 212 with neuropathy. ESM Table 2 shows the prevalence of these complications by DPP treatment arm. Figure 2a shows the distribution of microvascular complications at follow-up, including 37 participants with retinopathy and nephropathy, 49 with nephropathy and neuropathy, 17 with retinopathy and neuropathy, and four with all three complications.

Of the 353 metabolites included in this analysis, 105 were associated with one or more complication. Most compounds (74 metabolites; 70.5%) were associated with a single complication; 27 were shared between pairs of complications (six shared between retinopathy and nephropathy, ten shared between retinopathy and neuropathy, and 11 shared between nephropathy and neuropathy), and four were shared across all three complications (Fig. 2b). ESM Table 3 shows the 105 metabolite hits identified by LASSO. These metabolites were predominantly in pathways involved in metabolism of lipids (glycerophospholipids, glycerolipids, phospholipids/plasmalogens) or branched chain amino acids (keto acids, carboxylic acids, hydroxy acids), and/or involved in energy homeostasis processes and metabolism of cholesterol, steroids and fatty acids.

For the 69 metabolites that exhibited no interaction with DPP treatment arm, we ran multivariable models among all participants, adjusting for model 1 covariates plus treatment arm (ESM Tables 4–6). Of these metabolites, two were associated with one or more microvascular outcome: higher histidine at baseline was associated with lower odds of nephropathy (OR 0.75; 95% CI 0.69, 0.88), and higher baseline serine was associated with lower odds of nephropathy (OR 0.69; 95% 0.58, 0.82) and neuropathy (OR 0.68; 95% CI 0.56, 0.84) (Table 1). The direction and magnitude of these associations did not change after adjusting for mean HbA_1c_ during follow-up, incident diabetes or baseline eGFR (Table 1, models 2–4).

**Table 1:** Associations of circulating metabolites at baseline with prevalent microvascular complications at approximately 15 years of follow-up in pooled analysis of participants in the DPPOS

Compound	OR (95% CI)	Adjusted *p* value
Neuropathy		
Serine		
Model 1	0.68 (0.56, 0.84)	0.019*
Model 2	0.68 (0.56, 0.83)	
Model 3	0.68 (0.55, 0.83)	
Model 4	0.68 (0.56, 0.83)	
Nephropathy		
Histidine		
Model 1	0.75 (0.69, 0.88)	0.015*
Model 2	0.75 (0.64, 0.88)	
Model 3	0.74 (0.63, 0.87)	
Model 4	0.74 (0.63, 0.86)	
Serine		
Model 1	0.69 (0.58, 0.82)	0.001**
Model 2	0.71 (0.60, 0.83)	
Model 3	0.70 (0.59, 0.83)	
Model 4	0.70 (0.59, 0.83)	

No associations were identified for retinopathy

OR and 95% CI were calculated from a logistic regression model, weighted for the inverse propensity of having metabolomics data at baseline. Model 1 was adjusted for sex, race, time elapsed, baseline age, HbA_1c_, BMI and smoking status, and DPP treatment arm. Model 2 comprised the model 1 covariates plus mean HbA_1c_ during follow-up. Model 3 comprised the model 1 covariates plus incident diabetes. Model 4 comprised the model 1 covariates plus baseline eGFR

^*^Indicates *p*<0.05 after FDR correction; **indicates *p*<0.01 after FDR correction. Presented only for model 1 as this model was used to identify metabolites associated with outcomes

ESM Tables 7–9 show the results for model 1 for the 36 metabolites that exhibited an interaction with treatment arm. Table 2 shows the results for metabolites that were significant predictors of one or more outcome by treatment arm. In the metformin arm, each 1 SD increment in *N*-carbamoyl-β-alanine was associated with 1.99 (95% CI 1.38, 2.99) times the odds of nephropathy, whereas each 1 SD increment in C22:0-sphingomyelin (C22:0-SM) was associated with 0.54 (95% CI 0.37, 0.77) times the odds of neuropathy. In the lifestyle intervention arm, each 1 SD increment in quinolinic acid was associated with 1.64 (95% CI 1.24, 2.19) times the odds of neuropathy. None of the estimates changed appreciably after adjusting for covariates in models 2–4 (Table 2).

**Table 2:** Associations of circulating metabolites at baseline with prevalent microvascular complications at the 15-year follow-up, stratified by treatment arm, in the DPPOS

Compound	OR (95% CI)	Adjusted *p* value
Metformin		
C22:0-SM: neuropathy		
Model 1	0.54 (0.37, 0.77)	0.049*
Model 2	0.53 (0.37, 0.75)	
Model 3	0.54 (0.37, 0.78)	
Model 4	0.55 (0.38, 0.78)	
*N*-carbamoyl-β-alanine: nephropathy		
Model 1	1.99 (1.38, 2.99)	0.06
Model 2	1.95 (1.37, 2.90)	
Model 3	1.96 (1.37, 2.92)	
Model 4	1.85 (1.31, 2.73)	
Lifestyle		
Quinolinic acid: neuropathy		
Model 1	1.64 (1.24, 2.19)	0.049*
Model 2	1.65 (1.24, 2.21)	
Model 3	1.65 (1.24, 2.21)	
Model 4	1.65 (1.24, 2.21)	

No associations were identified for placebo

ORs and 95% CIs were calculated from a logistic regression model, weighted for the inverse propensity of having metabolomics data at baseline. Model 1 was adjusted for sex, race, time elapsed, baseline age, HbA_1c_, BMI and smoking status, and DPP treatment arm. Model 2 comprised the model 1 covariates plus mean HbA_1c_ during follow-up. Model 3 comprised the model 1 covariates plus incident diabetes. Model 4 comprised the model 1 covariates plus baseline eGFR

^*^Indicates *p*<0.05 after FDR correction. Presented only for model 1 as this model was used to identify metabolites associated with outcomes

ESM Table 10 shows the distribution of diabetes and microvascular complications. Although most occurrences of microvascular complications were observed in participants who developed diabetes (72.9% of individuals with nephropathy, 58.0% of those with neuropathy and 70.6% of those with retinopathy), a non-negligible proportion of participants with one or more microvascular complication did not have diabetes (27.1% of individuals with nephropathy, 42.0% of those with neuropathy, and 29.4% of those with retinopathy).

The results of sensitivity analyses performed after excluding 107 participants who developed one or more complication are described in ESM Results and shown in ESM Tables 11 and 12, and results for the 952 participants who developed diabetes are shown in ESM Fig. 1 and ESM Tables 13 and 14.

## Discussion

In this study of 1947 adults at high risk of diabetes, we identified metabolite profiles that were prospectively associated with prevalent retinopathy, nephropathy and neuropathy approximately 15 years later. We initially identified 105 metabolites that predicted at least one microvascular complication; the majority (70%) of these were linked to a single outcome. After further investigating metabolite–outcome associations while considering the heterogeneity of effects by DPP treatment group, we found that, among all participants (irrespective of treatment arm), higher circulating baseline histidine was associated with lower odds of nephropathy, and higher baseline serine predicted lower odds of nephropathy and neuropathy. In the metformin arm, higher baseline *N*-carbamoyl-β-alanine was associated with greater odds of nephropathy, and higher baseline C22:0 SM was related to lower odds of neuropathy. In the lifestyle intervention arm, higher quinolinic acid was associated with greater odds of neuropathy. These associations were not explained by diabetes development, mean HbA_1c_ during follow-up, or kidney function.

### Pooled analysis for metabolites that exhibited no heterogeneity of effects by treatment arm

Among all participants, circulating baseline histidine was inversely associated with prevalent nephropathy at follow-up. Histidine is an essential, anti-inflammatory amino acid that is involved in protein synthesis, tissue repair, growth and blood cell generation [19], and has antioxidant properties via its role in metal ion chelation and sequestration of advanced glycation end-products [20]. Our findings align with longstanding literature documenting lower circulating histidine among individuals with chronic kidney disease [21] and uraemia [22], and newer evidence showing that histidine supplementation protects against oxidative damage to nephrons in the context of chronic kidney disease [23]. The findings from the present study extend the literature by demonstrating that lower circulating histidine is prospectively associated with greater odds of nephropathy—possibly over a decade in advance—even among individuals who have not yet developed diabetes.

We also found an inverse relationship of baseline serine with nephropathy and neuropathy at follow-up. Serine is a non-essential amino acid that is involved in protein synthesis, one-carbon and glycine metabolism and neurotransmitter production [24]. Inadequate intake of l-serine has been implicated in impaired nervous system function owing to abnormal phospholipid and sphingolipid metabolism/synthesis [25]. Our finding of a protective effect of serine aligns with prior DPPOS work reporting an inverse association of serine with incidence of diabetes [9, 18], and suggests that lower circulating serine may be a biomarker of nephropathy and neuropathy risk even in the absence of diabetes (given that 27.1% of participants with nephropathy and 42.0% of participants with neuropathy did not develop diabetes during follow-up). Further, l-serine supplementation may be a possible intervention strategy to halt or slow progression of these microvascular complications.

### Stratified analysis for metabolites that exhibited heterogeneity of effects by DPP treatment arm

Among participants randomised to metformin, higher baseline *N*-carbamoyl-β-alanine was associated with greater odds of nephropathy. *N*-Carbamoyl-β-alanine is an intermediate of uracil metabolism [26], with higher levels of this metabolite being indicative of protein carbamylation, a post-translational modification that is upregulated as kidney function declines and with accumulation of urea [27]. Accordingly, the association of this metabolite with nephropathy may reflect subclinical progression of nephropathy that may have already been present at baseline.

Within the metformin arm, we also observed an inverse association between baseline C22:0 SM and neuropathy. These findings are in contrast to those of a recent study in 23 Japanese young adults, which noted higher plasma levels of this sphingomyelin as a correlate of obesity and metabolic syndrome parameters [28]. However, C22:0 SM is functionally related to docosanoic acid, another 22-carbon long-chain fatty acid that was inversely related to incident diabetes in the Nurses’ Health Study and the Health Professionals Follow-up Study [29]. Similarly, docosanoic acid was inversely related to HbA_1c_ in a feeding trial of 111 middle-aged adults with diabetes [30]. In the DPPOS, we previously found an inverse association of baseline plasma C22:1 SM, which has a similar structure to C22:0 SM, with diabetes risk [18]. We posit that while the physiological effects of C22:0 SM require further investigation, this metabolite may confer protective effects for neuropathy via chemical properties shared with docosanoic acid and C22:1 SM.

### Lifestyle intervention arm

Among participants in the lifestyle intervention arm, higher baseline quinolinic acid, an endogenous neurotoxin generated from the kynurenine pathway [31], was associated with greater odds of neuropathy. This finding aligns with those in rodent models showing that administration of quinolinic acid at the base of the cerebral hemisphere activated peroxisome proliferator-activated receptor gamma (PPARγ), which led to neuronal degeneration [32]. Upregulation of the kynurenine pathway has also been implicated in insulin resistance and poor glycaemic management among individuals with [33] and without [34] diabetes, suggesting that the association we observed reflects early pathophysiological processes underlying the parallel progression of neuropathy and diabetes.

### Comparison of findings with current literature

This analysis identified several pathways that are relevant to the pathophysiology of diabetes and microvascular complications, including those related to metabolism of lipids (glycerophospholipids, glycerolipids, phospholipids/plasmalogens) and branched chain amino acids (keto acids, carboxylic acids, hydroxy acids), as well as compounds reflecting energy homeostasis processes and metabolism of cholesterol, steroids and fatty acids. These findings align with a recent scoping review of articles on the metabolite profiles of diabetes-related microvascular complications [35], which included studies of rodent models and human studies (primarily small case–control studies, mostly with fewer than 150 participants) comparing the plasma, serum or urine metabolite profiles of humans.

Unexpectedly, in the main analysis, we did not find any metabolites that were associated with retinopathy, which is considered to be the most diabetes-specific complication of the three outcomes assessed in this study. In a case–control study that conducted targeted metabolomics assays for serum tryptophan and its metabolites, Munipally et al [36] identified elevated levels of indoleamine 2,3-dioxygenase and kynurenines among participants with diabetic retinopathy (*n*=46) vs control individuals (*n*=35). However, this study measured metabolites concurrently with case and control status (having retinopathy or not), as opposed to prospectively as done in the present study. In the Finnish METSIM cohort, Fernandes Silva et al [37] identified 17 metabolites in amino acid, fatty acid and lipid/sphingolipid pathways that were associated with incident retinopathy during 12 years’ follow-up. In the present analysis, several of these pathways were captured by metabolite hits for retinopathy in the initial feature selection step (i.e. tryptophan and carnitines related to amino acid and lipid metabolism) but did not survive FDR correction. There are also noteworthy differences between the METSIM study and the DPPOS that probably contributed to their ability to detect differences in these metabolites in relation to retinopathy. Namely, the METSIM study included only men with type 2 diabetes, and participants had a markedly higher baseline HbA_1c_ than those in the DPPOS (54 mmol/mol [7.1%] and 45 mmol/mol [6.3%], respectively, for individuals with retinopathy and those without, vs 41 mmol/mol [5.9%] in the DPPOS).

We noted that among the metabolites that exhibited heterogeneity of effects by DPP treatment arm, such effects were observed among those randomised to metformin and the lifestyle intervention but not in the placebo group. This may be due to long-term effects of the interventions, such as the long-lasting effects of metformin on metabolic parameters [38], effects of the lifestyle intervention on subsequent health behaviours and hyperglycaemia progression [10], and unmeasured confounding or unaccounted for changes to lifestyle or health history during follow-up.

### Comparison of main findings with those of sensitivity analyses

To rule out the possibility that overlaps in metabolite profiles across microvascular complications primarily reflected the occurrence of multiple complications in the same individual, we excluded 107 participants who developed more than one complication. In this sub-sample also, the majority of hits in the metabolite selection step were linked to a single complication. While we did not identify associations of any metabolites with microvascular outcomes for metabolites assessed via pooled analysis, C9 carnitine in the metformin arm and serine in the lifestyle intervention arm were each inversely related to neuropathy. These compounds are on lipid (fatty acyls for C9 carnitine) and amino acid (serine) pathways that are similar to those for metabolites identified for neuropathy in the main analysis, i.e. C22:0 SM, a sphingolipid, in the metformin arm, and quinolinic acid, in the branched chain amino acid pathway, in the lifestyle intervention arm, thus supporting the consistency of our findings.

To better ensure that the metabolites of interest are relevant to hyperglycaemia, we conducted a second sensitivity analysis that was restricted to participants who developed diabetes during follow-up. As with the main analysis, most metabolite hits were specific to a single complication. In subsequent multivariable models for metabolites that did not exhibit heterogeneity of effects of treatment arm and were thus assessed in pooled analysis, baseline C22:0 SM, serine, histidine and the plasmalogen C36:1 phosphatidylcholine were each associated with greater odds of nephropathy, and baseline 1-methylhistamine and the triacylglycerol C56:1 were associated with greater odds of nephropathy. The inverse associations of histidine and serine with nephropathy irrespective of treatment arm align with the main results. However, in contrast to the main results, we did not identify any metabolite predictors of neuropathy. Further, whereas the main analysis did not identify any predictors of retinopathy after FDR correction, C26 carnitine was significantly associated with retinopathy in this sub-sample, probably reflecting the specificity of retinopathy to diabetes and the greater power to detect this association among participants who developed diabetes. For metabolites that exhibited an interaction with treatment arm, we did not observe associations of metabolites with any outcome in stratified analysis. However, we noted a marginal association of quinolinic acid with neuropathy with the lifestyle intervention arm (FDR-corrected *p* value = 0.06), as in the main analysis, again supporting the consistency of our results.

Differences in findings for the main vs sensitivity analyses are expected given differences in physiology among individuals with diabetes vs those without, and those who develop one vs multiple complications, in addition to the number of comparisons made and varying FDR *p* value thresholds for statistical significance. Despite the above, we note important consistencies. First, histidine was inversely associated with greater odds of nephropathy at follow-up irrespective of treatment arm, suggesting that low levels of this metabolite should be explored further as a biomarker of nephropathy risk. Second, the metabolites of interest were consistently and most frequently found in amino acid, lipid, fatty acid and energy homeostasis pathways. Finally, the majority of metabolites of interest – both in the initial feature selection step as well as subsequent steps – were specific to a single complication, suggesting that the three microvascular complications may arise from distinct pathways.

### Strengths and limitations

Strengths of this study include broad racial/ethnic representation and the ability to assess metabolite–microvascular outcome associations among individuals who are at high risk of diabetes but did not yet have diabetes at the time of metabolite assessment.

This study also has several limitations. First, we did not assess participants for complications at baseline, so we cannot be certain that microvascular complications were not already present at the time that the metabolites were measured. Regardless, associations of metabolites with the complications over time remain relevant to understanding pathophysiology. Second, despite extensive sensitivity analyses, we cannot rule out the possibility that shared metabolite profiles reflect the existence of multiple complications within the same individual or be certain that the metabolite profiles identified herein reflect diabetes-related pathways. Third, despite employing multiple techniques to mitigate type 1 error, the possibility of false-positive findings remains a concern. Conversely, false-negative findings are also a possibility. While a balance between the two is ideal, we posit that inflation of false-positive findings will more quickly erode the credibility of the findings – especially in high-dimensional analyses for which ‘hits’ are reported – and hinder future replication efforts. Fourth, we did not perform sex-stratified analysis given that sex differences in metabolite profiles were not part of our a priori research question and there may therefore be differences in the pathophysiology and mechanisms of diabetes complications for men vs women that were not detected in this study. However, we view this analysis as an exploratory first step to understanding heterogeneity in pathways underlying diabetes complications that future studies may expand on through subgroup analyses. Finally, as we did not include a validation population, generalisability may be limited.

### Conclusions

Metabolite profiling of plasma from 1947 middle-aged adults at high risk of developing type 2 diabetes revealed novel metabolite profiles for microvascular complications over 15 years’ follow-up, independent of diabetes development and HbA_1c_. Most metabolites were specific to a single complication, suggesting that, while all three microvascular complications can arise from poorly managed hyperglycaemia and oxidative stress, they may also have distinct pathophysiological mechanisms.

We suggest that there is a need to not only monitor HbA_1c_ but also other putative metabolomic biomarkers to halt the progression of microvascular complications. Our findings also indicate that it may be important to identify biomarkers of microvascular complications among individuals at risk for diabetes who do not yet meet diagnostic criteria. Further, given known racial/ethnic and sex differences in the aetiology of diabetes and its complications [39], future studies should explore differences across sociodemographic subgroups to inform strategies for prevention, management and prognosis of diabetes-associated complications.

## Supplementary Information

Below is the link to the electronic supplementary material.

Supplementary file1 (PDF 1.23 MB)

## Data Availability

In accordance with the NIH Public Access Policy, we continue to provide all manuscripts to PubMed Central including this manuscript. DPP/DPPOS have provided the protocols and lifestyle and medication intervention manuals to the public through its public website (https://www.dppos.org). The DPPOS abides by the National Institute of Diabetes and Digestive and Kidney Disease (NIDDK) data sharing policy and implementation guidance as required by the NIH/NIDDK (https://repository.niddk.nih.gov/studies/dppos/).

## Abbreviations

C22:0-SM: C22:0-sphingomyelin
DPP: Diabetes Prevention Program
DPPOS: Diabetes Prevention Program Outcomes Study
FDR: False discovery rate
LASSO: Least absolute shrinkage and selection operator
