# Supplementary material for: Shared and distinct metabolomics profiles associated with microvascular complications in the Diabetes Prevention Program Outcomes Study
Source: Diabetologia. Author manuscript; Available in PMC 2026 Mar 3. (PMC12685977; doi:10.1007/s00125-025-06571-8)
Supplement: Electronic Supplemental Materials [file NIHMS2128138-supplement-Electronic_Supplemental_Materials.pdf]

ELECTRONIC SUPPLEMENTAL MATERIAL (ESM)

ESM Methods

Metabolomics profiling

For the assessment of amino acids and amines, hydrophilic interaction chromatography (Waters; Milford, MA) was coupled to a Q Exactive mass spectrometer (MS) in positive ion mode (Thermo Fisher Scientific, Waltham, MA). For the assessment of lipids, C8 chromatography (Waters) was coupled to a Q Exactive MS in positive ion mode. For the assessment of organic acids, amide chromatography (Waters, Milford, MA) was coupled to an Agilent 6490 triple quadrupole MS (Agilent Technologies, Santa Clara, CA) using negative ion mode electrospray ionization. Quality control measures included monitoring of isotope-labeled internal standards in each sample and the use of pooled plasma reference samples inserted every 10–20 study samples, to serve as a reference to standardize within and across batches. Separate pooled plasma injections were included every 20 injections to gauge the effectiveness of normalization, and to determine the coefficient of variation of each metabolite. Of the 365 metabolites measured, we restricted the present analysis to 353 metabolites with missingness <20%.

ESM Results

Results of sensitivity analyses

First, we excluded the 107 individuals who had more than one complication at follow-up (187 cases of nephropathy, 136 cases of retinopathy, and 142 cases of neuropathy). The BOLASSO in Step 1 identified 109 metabolite hits, for which the majority (n=85; 76%) were specific to a single complication, 23 were shared between pairs of complications, and 3 were shared across all three (**ESM Figure 1**). We noted moderate overlap in metabolite identities with those identified in Step 1 for the main analysis, as indicated by the bolded compounds in **ESM Table 11**. In Step 2, for metabolites assessed in pooled analysis, we did not identify any significant metabolite predictors of any microvascular outcome after FDR correction. For

metabolites assessed via stratified analysis, C9 carnitine and serine were inversely related neuropathy in the metformin and lifestyle arms, respectively, after FDR correction (**ESM Tables 12**).

Second, we restricted the analysis to the 952 participants who developed diabetes during follow-up to ensure that the complications reflect diabetes-associated complications. In Step 1, the BOLASSO identified 89 metabolites that was associated with least 1 microvascular complication. As with the primary and supplementary analyses we have previously run, most metabolite hits (52%) were specific to a single complication (26 for retinopathy, 21 for neuropathy, and 25 for nephropathy), 13 were shared across pairs of complications, and 4 were shared across all three complications. The list of metabolite hits are now shown in **ESM Table 13**. In Step 2, 22 metabolites that exhibited an interaction by DPP treatment arm and 67 metabolites did not. For the 22 metabolites that exhibited an interaction with treatment arm, we ran multivariable regression models separately by treatment arm. None of the metabolites were associated with any outcomes after FDR correction, therefore no further analyses were run for these compounds. For the 67 compounds that did not exhibit an interaction with DPP treatment arm, the pooled multivariable analysis yielded 7 hits after FDR correction. For these compounds, we ran the sequence of multivariable models. These results are in **ESM Table 14**.

ESM Table 1 Baseline characteristics of 1,947 participants in the Diabetes Prevention Program Outcome Study (DPPOS).

	Mean ± SD or N (%)				
	Overall<br>*n* = 1,947	Lifestyle<br>*n* = 665	Metformin<br>*n* = 647	Placebo<br>*n* = 635	*P*-value[a]
Age, years	53.1 ± 10.1	53.3 ± 10.8	53.6 ± 9.7	52.4 ± 9.8	0.08
Sex					<0.001
Male	649 (33.3%)	234 (35.2%)	245 (37.9%)	170 (26.8%)	
Female	1,298 (66.7%)	431 (64.8%)	402 (62.1%)	465 (73.2%)	
Race and ethnicity					0.15
Non-Hispanic White	1,104 (56.7%)	376 (56.5%)	382 (59.0%)	346 (54.5%)	
Black or African American	370 (19.0%)	115 (17.3%)	125 (19.3%)	130 (20.0%)	
Hispanic	256 (13.1%)	88 (13.2%)	83 (12.8%)	85 (13.0%)	
American Indian	124 (6.4%)	46 (6.9%)	29 (4.5%)	49 (7.7%)	
Asian	93 (4.8%)	40 (6.0%)	28 (4.3%)	25 (3.9%)	
Smoking status at enrollment					0.13
Never	1,108 (57%)	386 (58.0%)	369 (57.0%)	353 (56.0%)	
Current	120 (6.2%)	29 (4.4%)	41 (6.3%)	50 (7.9%)	
Former	719 (37.0%)	250 (38.0%)	237 (37.0%)	232 (37.0%)	
Body mass index, kg/m^2	32.3 ± 6.5	32.1 ± 6.5	32.0 ± 6.5	32.9 ± 6.7	0.008
Systolic blood pressure, kPa	16.6 ± 2.0	16.5 ± 2.0	16.7 ± 2.0	16.5 ± 2.0	0.30
Diabolic blood pressure, kPa	10.4 ± 1.2	10.4 ± 1.2	10.4 ± 1.3	10.4 ± 1.2	0.90
Hemoglobin A_{1c} (HbA_{1c}), mmol/mol	41.2 ± 5.4	41.1 ± 5.5	41.3 ± 5.4	41.1 ± 5.3	0.80
HbA_{1c}, (%)	5.9 ± 0.5	5.9 ± 0.5	5.9 ± 0.5	5.9 ± 0.5	0.80
Time since enrollment, years	12.7 ± 2.9	12.7 ± 3.0	12.8 ± 2.9	12.8 ± 2.8	>0.99
Fasting glucose, mmol/l	5.9 ± 0.5	5.9 ± 0.5	5.9 ± 0.5	5.9 ± 0.5	0.90
Diabetes at follow-up	1,163 (59.7%)	387 (58.2%)	383 (59.2%)	393 (61.9%)	0.40

[a] Kruskal-Wallis rank sum test for continuous variables; Pearson's Chi-squared test for categorical and ordinal variables

ESM Table 2 Distribution of microvascular complications cases stratified by original Diabetes Prevention Program treatment arm among 683 participants who had at least one complication at ~15 y of follow-up.

	Nephropathy	Retinopathy	Neuropathy
Placebo (*n* = 213)	81	57	75
Metformin (*n* = 252)	108	72	72
Lifestyle (*n* = 218)	88	65	65

ESM Table 3 Metabolite hits at baseline (*n* = 105 compounds) that predict each microvascular complication at ~15 y follow-up among 1,947 participants in the Diabetes Prevention Program Outcomes Study (DPPOS).[a]

Neuropathy		*Nephropathy*		*Retinopathy*	
Metabolite name	Class	Metabolite name	Class	Metabolite name	Class
DMGV	Keto acids & derivatives	DMGV	Keto acids & derivatives	3-Aminoisobutyric acid	Carboxylic acids & derivatives
2-Hydroxybutyric acid	Hydroxy acids & derivatives	3-Aminoisobutyric acid	Carboxylic acids & derivatives	Bilirubin	Tetrapyrroles & derivatives
3-Hydroxybutyric acid	Hydroxy acids & derivatives	Allantoin	Azoles	Deoxycholic acid	Steroids & steroid derivatives
Anthranilic acid	Aminobenzoic acids	Anthranilic acid	Aminobenzoic acids	D Gluconic acid	Organooxygen compounds
Bilirubin	Tetrapyrroles & derivatives	Citrulline	Carboxylic acids & derivatives	Hippuric acid	Benzene & substituted derivatives
Deoxycholic acid	Steroids & steroid derivatives	Deoxycholic acid	Steroids & steroid derivatives	Hydroxyphenylpyruvic acid	Benzene & substituted derivatives
Gluconic acid	Organooxygen compounds	Glutathione	Carboxylic acids & derivatives	Indole 3 carboxylic acid	Indoles & derivatives
Hippuric acid	Benzene & substituted derivatives	Homogentisic acid	Benzene & substituted derivatives	Inosine	Purine nucleosides
Lactic acid	Benzene & substituted derivatives	Keto-isocaproic acid	Benzene & substituted derivatives	Succinic acid	Carboxylic acids & derivatives
Malic acid	Hydroxy acids & derivatives	Orotic acid	Diazines	Uridine	Pyrimidine nucleosides
Malonic acid	Carboxylic acids & derivatives	Oxalic acid	Carboxylic acids & derivatives	Xanthosine	Purine nucleosides
Oxalic acid	Carboxylic acids & derivatives	Oxaloacetic acid	Keto acids & derivatives	C36:3 PS Plasmalogen	Plasmalogen phospholipids
Quinolinic acid	Pyridines & derivatives	Pyruvic acid	Carboxylic acids & derivatives	C36:0 PE	Glycerophospholipids
Taurocholic acid	Steroids & steroid derivatives	Uric acid	Imidazopyrimidines	C38:4 PE	Glycerophospholipids
C40:10 PC	Glycerophospholipids	Xanthosine	Purine nucleosides	C34:1 PI	Glycerophospholipids
C32:0 PE	Glycerophospholipids	C34:4-PC Plasmalogen	Glycerophospholipids	C22:1 MAG	Glycerolipids
C34:1 PI	Glycerophospholipids	C36:5-PC Plasmalogen A	Glycerophospholipids	C36:0 DAG	Glycerolipids
C36:3 PS plasmalogen	Plasmalogen phospholipids	C36:1-PC Plasmalogen	Glycerophospholipids	C54:9 TAG	Glycerophospholipids
Sphingosine	Amines	C32:0-PE	Glycerophospholipids	Asparagine	Carboxylic acids & derivatives
C14:0 SM	Sphingolipids	C24:1 Ceramide D18:1	Sphingolipids	Arginine	Carboxylic acids & derivatives
C18:1 SM	Sphingolipids	C22:0-SM	Sphingolipids	Tryptophan	Indoles & derivatives
C18:0 CE	Steroids & steroid derivatives	C18:3-CE	Steroids & steroid derivatives	Hydroxyproline	Carboxylic acids & derivatives
C22:5 CE	Steroids & steroid derivatives	C22:4-CE	Steroids & steroid derivatives	Dimethylglycine	Carboxylic acids & derivatives
C32:2 DAG	Glycerolipids	C22:1-MAG	Glycerolipids	SDMA	Carboxylic acids & derivatives
C42:0 TAG	Triradylglycerols	C32:2-DAG	Glycerolipids	NMMA (NMMA)	Carboxylic acids & derivatives
Serine	Carboxylic acids & derivatives	C56:2-TAG	Glycerolipids	1-Methylhistamine	Organonitrogen compounds
Methionine	Carboxylic acids & derivatives	Glycine	Carboxylic acids & derivatives	Niacinamide	Pyridines & derivatives
Aspartate	Carboxylic acids & derivatives	Serine	Carboxylic acids & derivatives	Phosphocholine	Organonitrogen compounds
Hydroxyproline	Carboxylic acids & derivatives	Methionine	Carboxylic acids & derivatives	Alpha-glycerophosphocholine	Glycerophospholipids
Citrulline	Carboxylic acids & derivatives	Histidine	Carboxylic acids & derivatives	Xanthine	Purine nucleosides
Dimethylglycine	Carboxylic acids & derivatives	Phenylalanine	Carboxylic acids & derivatives	Pipecolic acid	Carboxylic acids & derivatives
ADMA	Carboxylic acids & derivatives	Proline	Carboxylic acids & derivatives	5-Aminolevulinic acid	Carboxylic acids & derivatives
1-Methylhistamine	Organonitrogen compounds	NMMA	Carboxylic acids & derivatives	Methionine sulfoxide	Carboxylic acids & derivatives
N-carbamoyl-beta-alanine	Amines	Kynurenic acid	Quinolines & derivatives	Sarcosine	Carboxylic acids & derivatives
Niacinamide	Pyridines & derivatives	1-Methylhistamine	Organonitrogen compounds	C2 carnitine	Fatty acyls
Phosphocholine	Organonitrogen compounds	N-carbamoyl-beta-alanine	Amines	C3 carnitine	Fatty acyls
Alpha-glycerophosphocholine	Glycerophospholipids	Thiamine	Diazines	C4-OH carnitine	Fatty acyls
Creatine	Carboxylic acids & derivatives	Betaine	Carboxylic acids & derivatives	C9 carnitine	Fatty acyls
Creatinine	Carboxylic acids & derivatives	Phosphocholine	Organonitrogen compounds	C18:1-OH carnitine	Fatty acyls
Cytosine	Diazines	Xanthosine	Purine nucleosides	C26 carnitine	Fatty acyls
Xanthosine	Purine nucleosides	Cotinine	Pyridines & derivatives		
Cotinine	Pyridines & derivatives	C2 carnitine	Fatty acyls		
Methionine sulfoxide	Carboxylic acids & derivatives	C5-DC carnitine	Fatty acyls		
Sarcosine	Carboxylic acids & derivatives	C7 carnitine	Fatty acyls		
Beta-alanine	Carboxylic acids & derivatives	C18:1-OH carnitine	Fatty acyls		
C4-OH carnitine	Fatty acyls	C26 carnitine	Fatty acyls		
C5 carnitine	Fatty acyls	Methylhydroxyisobutyric acid	Hydroxy acids & derivatives		
C7 carnitine	Fatty acyls	Methylthioadenosine	5'-deoxyribonucleosides		
C14 carnitine	Fatty acyls				
C14:2 carnitine	Fatty acyls				
C:16-OH carnitine	Fatty acyls				
Methylthioadenosine	5'-deoxyribonucleosides				

Abbreviations: ADMA: Asymmetric dimethylarginine; CE: cholesterol ester; DAG: Diacylglycerol; DMGV: Dimethylguanidino valeric acid; MAG: monoacylglycerol; NMMA: L-targinine; OH: hydroxyl group; PC: phosphatidylcholine; PE: phosphatidylethanolamine; PS: phosphatidylserine; SDMA: Symmetric dimethylarginine; TAG: triacylglycerol

[a] Selected from bootstrapped least absolute shrinkage and selection operator (BOLASSO) model adjusted for sex; race; time elapsed during follow-up; and baseline age, HbA_{1c}, BMI, and smoking status.

ESM Table 4 Odds ratios (OR) and 95% confidence intervals (CI) reflecting associations of circulating metabolites at baseline with nephropathy at ~15 y of follow-up in the Diabetes Prevention Program Outcomes Study (DPPOS).

Metabolite	OR[a]	95% CI	Adj. P-value
Serine	0.691	(0.582, 0.816)	0.0014***
Histidine	0.748	(0.686, 0.878)	0.02*
C18:3 CE	0.790	(0.672, 0.928)	0.10
C36:1 PC plasmalogen	0.801	(0.684, 0.938)	0.11
Oxalic acid	0.796	(0.657, 0.964)	0.28
C18:1 sm	0.825	(0.697, 0.976)	0.29
Methionine	0.830	(0.703, 0.981)	0.29
Cotinine	1.169	(1.006, 1.359)	0.36
Uridine	0.858	(0.731, 1.006)	0.39
Asparagine	0.861	(0.739, 1.002)	0.39
C32:0 PE	0.836	(0.693, 1.009)	0.39
Dimethylglycine	1.083	(0.992, 1.183)	0.44
Methyl hydroxyisobutyric	1.118	(0.984, 1.269)	0.46
Hydroxyproline	1.116	(0.982, 1.268)	0.46
Homogentisic acid	1.116	(0.973, 1.279)	0.54
Thyroxine	1.114	(0.960, 1.293)	0.60
Malic acid	0.893	(0.761, 1.048)	0.60
Beta alanine	0.892	(0.757, 1.051)	0.60
C14:2 carnitine	0.882	(0.745, 1.044)	0.60
C24:1 ceramide D18:1	1.114	(0.959, 1.295)	0.60
Tryptophan	0.899	(0.761, 1.063)	0.62
Phosphocholine	0.887	(0.734, 1.070)	0.62
Sphingosine	0.822	(0.602, 1.121)	0.62
Betaine	0.898	(0.759, 1.061)	0.62
1-Methylhistamine	1.075	(0.951, 1.214)	0.63
Methylthioadenosine	0.893	(0.738, 1.081)	0.63
Thiamine	1.095	(0.944, 1.270)	0.63
Deoxycholic acid	1.085	(0.939, 1.254)	0.66
C36:4 PC plasmalogen	0.915	(0.779, 1.075)	0.66
D-gluconic acid	1.071	(0.934, 1.229)	0.75
Bilirubin	0.931	(0.790, 1.097)	0.79
Succinic acid	0.892	(0.672, 1.183)	0.79
C34:1 PI	0.935	(0.789, 1.109)	0.79
C36:3 PS plasmalogen	1.057	(0.914, 1.222)	0.79
C42:0 TAG	1.062	(0.931, 1.212)	0.79
Aspartate	0.939	(0.797, 1.106)	0.79
Creatine	1.071	(0.916, 1.252)	0.79
C5 carnitine	1.062	(0.918, 1.228)	0.79
C14 carnitine	0.932	(0.793, 1.096)	0.79
C34:4 PC plasmalogen	0.937	(0.798, 1.101)	0.79
Inosine	1.057	(0.904, 1.234)	0.80
C36:0 DAG	1.047	(0.911, 1.204)	0.80
5-Aminolevulinic acid	1.039	(0.921, 1.172)	0.80
Glutathione reduced	0.931	(0.751, 1.152)	0.80
C22:4 CE	0.949	(0.804, 1.120)	0.80
Phenylalanine	1.050	(0.902, 1.224)	0.80
C18:0 CE	0.958	(0.822, 1.116)	0.85
Taurocholic acid	1.037	(0.905, 1.189)	0.86
C22:5 CE	0.966	(0.824, 1.133)	0.91
Orotic acid	0.959	(0.795, 1.157)	0.91
Pyruvic acid	0.965	(0.826, 1.127)	0.91
Pipecolic acid	1.028	(0.897, 1.177)	0.91
Cytosine	0.965	(0.806, 1.156)	0.91
Anthranilic acid	0.974	(0.834, 1.138)	0.95
Indole-3-carboxylic acid	1.024	(0.874, 1.199)	0.96
Oxaloacetic acid	0.975	(0.817, 1.164)	0.96

ESM Table 4 (continued) Odds ratios (OR) and 95% confidence intervals (CI) reflecting associations of circulating metabolites at baseline with nephropathy at ~15 y of follow-up in the Diabetes Prevention Program Outcomes Study (DPPOS).

Metabolite	OR[a]	95% CI	Adj. P-value
Hydroxyphenylpyruvic acid	0.985	(0.839, 1.156)	0.97
Xanthine	0.981	(0.835, 1.152)	0.97
Methionine sulfoxide	1.013	(0.865, 1.186)	0.97
C3 carnitine	1.013	(0.881, 1.165)	0.97
Proline	1.015	(0.872, 1.181)	0.97
Kynurenic acid	1.016	(0.871, 1.185)	0.97
Niacinamide	1.011	(0.858, 1.191)	0.97
Allantoin	1.010	(0.865, 1.178)	0.97
C38:4 PE	1.004	(0.860, 1.173)	0.99
3-Hydroxybutyric acid	0.995	(0.841, 1.178)	0.99
C32:2 DAG	0.995	(0.856, 1.156)	0.99
Alpha-glycerophosphocholine	1.001	(0.864, 1.159)	0.99
C7 carnitine	1.000	(0.873, 1.145)	0.99

Abbreviations**:** CE: cholesterol ester; DAG: Diacylglycerol; PC: phosphatidylcholine; PE: phosphatidylethanolamine; TAG: triacylglycerol

* Indicates P <0.05 and *** indicates P<0.001 after false discovery rate (FDR) correction.

a From a logistic regression model weighted for the inverse propensity of having metabolomics data at baseline, and adjusted for sex; race; time elapsed; baseline age, HbA_{1c}, BMI, and smoking status.

ESM Table 5 Odds ratios (OR) and 95% confidence intervals (CI) reflecting associations of circulating metabolites at baseline with neuropathy at ~15 y of follow-up in the Diabetes Prevention Program Outcomes Study (DPPOS).

Metabolite	OR[a]	95% CI	Adj. P-value
Serine	0.681	(0.555, 0.837)	0.02*
Uridine	0.795	(0.663, 0.954)	0.35
3-Hydroxybutyric acid	1.184	(1.010, 1.389)	0.35
C36:3 PS plasmalogen	1.161	(1.006, 1.340)	0.35
C18:1 SM	0.808	(0.663, 0.985)	0.35
Creatine	0.810	(0.673, 0.975)	0.35
Beta alanine	1.206	(1.015, 1.433)	0.35
C14 carnitine	1.210	(1.028, 1.424)	0.35
Bilirubin	1.157	(1.000, 1.338)	0.39
Anthranilic acid	0.750	(0.547, 1.029)	0.40
Oxalic acid	1.114	(0.986, 1.259)	0.40
Taurocholic acid	1.110	(0.985, 1.250)	0.40
C18:0 CE	0.845	(0.707, 1.010)	0.40
Methylthioadenosine	1.155	(0.985, 1.355)	0.40
C22:4 CE	0.822	(0.669, 1.009)	0.40
Malic acid	1.152	(0.973, 1.364)	0.41
C42:0 TAG	1.119	(0.980, 1.278)	0.41
Cytosine	0.836	(0.667, 1.049)	0.47
Cotinine	1.154	(0.954, 1.396)	0.51
C32:0 PE	1.133	(0.955, 1.344)	0.53
C34:1 PI	1.101	(0.948, 1.279)	0.69
Dimethylglycine	0.622	(0.290, 1.337)	0.70
Kynurenic acid	0.899	(0.754, 1.073)	0.72
Methionine sulfoxide	1.105	(0.927, 1.318)	0.73
C3 carnitine	0.873	(0.674, 1.132)	0.73
Methionine	0.907	(0.755, 1.091)	0.73
C7 carnitine	0.913	(0.771, 1.081)	0.73
Glutathione reduced	0.877	(0.686, 1.121)	0.73
Methyl-hydroxyisobutyric acid	0.911	(0.763, 1.088)	0.73
Tryptophan	0.911	(0.753, 1.101)	0.76
C5 carnitine	0.919	(0.771, 1.095)	0.76
C18:3 CE	0.927	(0.778, 1.106)	0.87
Sphingosine	0.877	(0.634, 1.214)	0.90
Asparagine	0.936	(0.789, 1.110)	0.91
D gluconic acid	0.928	(0.751, 1.146)	0.93
Succinic acid	1.056	(0.910, 1.224)	0.93
Phosphocholine	0.939	(0.783, 1.126)	0.93
Deoxycholic acid	1.019	(0.862, 1.205)	0.95
Hydroxyphenylpyruvic acid	1.035	(0.888, 1.208)	0.95
Indole 3 carboxylic acid	1.037	(0.861, 1.249)	0.95
Xanthine	1.022	(0.853, 1.225)	0.95
C36:4 PC plasmalogen	0.971	(0.815, 1.158)	0.95
C38:4 PE	1.027	(0.866, 1.218)	0.95
C36:0 DAG	1.018	(0.868, 1.193)	0.95
1-Methylhistamine	0.960	(0.819, 1.125)	0.95
Niacinamide	1.027	(0.861, 1.225)	0.95
Pipecolic acid	0.971	(0.803, 1.173)	0.95
5-Aminolevulinic acid	0.965	(0.800, 1.163)	0.95
C22:5 CE	1.049	(0.875, 1.257)	0.95
Alpha-glycerophosphocholine	0.949	(0.798, 1.130)	0.95
C14:2 carnitine	0.965	(0.802, 1.162)	0.95
Orotic acid	0.977	(0.802, 1.190)	0.95
Oxaloacetic acid	0.963	(0.782, 1.187)	0.95
Pyruvic acid	0.958	(0.805, 1.141)	0.95
C24:1 Ceramide D18:1	1.043	(0.884, 1.229)	0.95
Histidine	0.979	(0.826, 1.160)	0.95

ESM Table 5 (continued) Odds ratios (OR) and 95% confidence intervals (CI) reflecting associations of circulating metabolites at baseline with neuropathy at ~15 y of follow-up in the Diabetes Prevention Program Outcomes Study (DPPOS).

Metabolite	OR[a]	95% CI	Adj. P-value
Phenylalanine	0.953	(0.804, 1.129)	0.95
Proline	1.020	(0.855, 1.218)	0.95
Thiamine	1.054	(0.893, 1.243)	0.95
Betaine	0.975	(0.808, 1.177)	0.95
Inosine	0.981	(0.811, 1.186)	0.95
Thyroxine	0.984	(0.826, 1.173)	0.96
Hydroxyproline	0.989	(0.830, 1.177)	0.98
C34:4 PC plasmalogen	0.990	(0.837, 1.171)	0.98
C32:2 DAG	1.004	(0.860, 1.171)	0.98
Aspartate	1.005	(0.861, 1.174)	0.98
Allantoin	1.002	(0.852, 1.179)	0.98
Homogentisic acid	1.002	(0.847, 1.186)	0.98
C36:1 PC plasmalogen	0.996	(0.840, 1.182)	0.98

Abbreviations**:** CE: cholesterol ester; DAG: Diacylglycerol; MAG: monoacylglycerol; OH: hydroxyl group; PC: phosphatidylcholine; PE: phosphatidylethanolamine; PS: Phosphatidylserine; TAG: triacylglycerol

***** Indicates P <0.05 after false discovery rate (FDR) correction.

[a] From a logistic regression model weighted for the inverse probability having metabolomics data at baseline and adjusted for sex; race; time elapsed; baseline age, HbA_{1c}, BMI, and smoking status.

ESM Table 6 Odds ratios (OR) and 95% confidence intervals (CI) reflecting associations of circulating metabolites at baseline with retinopathy at ~15 y of follow-up in the Diabetes Prevention Program Outcomes Study (DPPOS).

Metabolite	OR[a]	95% CI	Adj. P-value[b]
Inosine	0.593	(0.331, 1.061)	0.58
Uridine	0.838	(0.701, 1.001)	0.58
C36:0 DAG	1.131	(0.987, 1.296)	0.58
Asparagine	1.209	(1.022, 1.431)	0.58
Dimethylglycine	0.395	(0.165, 0.945)	0.58
Thyroxine	1.213	(1.024, 1.436)	0.58
Methionine sulfoxide	1.166	(0.989, 1.376)	0.58
C3 carnitine	1.151	(1.004, 1.318)	0.58
Cytosine	1.161	(0.981, 1.375)	0.58
Orotic acid	1.130	(0.989, 1.292)	0.58
Xanthine	0.843	(0.691, 1.030)	0.60
Serine	1.151	(0.969, 1.367)	0.63
Pipecolic acid	1.109	(0.970, 1.268)	0.68
Deoxycholic acid	1.109	(0.941, 1.305)	0.68
D Gluconic acid	1.098	(0.963, 1.251)	0.68
Hydroxyproline	1.095	(0.952, 1.260)	0.68
1-Methylhistamine	1.084	(0.948, 1.238)	0.68
Niacinamide	0.869	(0.688, 1.099)	0.68
C32:0 PE	1.144	(0.956, 1.369)	0.68
C18:1 SM	0.894	(0.740, 1.081)	0.68
C42:0 TAG	0.858	(0.693, 1.062)	0.68
C14 carnitine	0.890	(0.731, 1.083)	0.68
Methylthioadenosine	0.869	(0.688, 1.098)	0.68
Homogentisic acid	1.108	(0.953, 1.289)	0.68
C18:3 CE	1.118	(0.938, 1.332)	0.68
Oxaloacetic acid	0.873	(0.690, 1.105)	0.68
C34:1 PI	0.892	(0.723, 1.102)	0.69
Creatine	1.102	(0.919, 1.321)	0.69
Allantoin	0.902	(0.743, 1.096)	0.69
Pyruvic acid	0.911	(0.767, 1.083)	0.69
Tryptophan	1.093	(0.906, 1.319)	0.78
Succinic acid methylmalon	0.861	(0.611, 1.213)	0.79
Methionine	1.073	(0.899, 1.279)	0.79
Alpha glycerophosphocholine	0.927	(0.773, 1.111)	0.79
Beta alanine	0.926	(0.771, 1.112)	0.79
C34:4 PC plasmalogen	1.072	(0.905, 1.271)	0.79
Phenylalanine	1.072	(0.902, 1.274)	0.79
Proline	0.924	(0.774, 1.104)	0.79
C36:4 PC plasmalogen	0.937	(0.778, 1.127)	0.82
5-Aminolevulinic acid	0.914	(0.711, 1.175)	0.82
3-Hydroxybutyric acid	0.941	(0.770, 1.150)	0.82
Oxalic acid	0.943	(0.782, 1.137)	0.82
Taurocholic acid	1.047	(0.923, 1.188)	0.82
C36:3 PS plasmalogen	1.052	(0.890, 1.244)	0.82
C32:2 DAG	0.943	(0.775, 1.147)	0.82
Cotinine	1.063	(0.875, 1.292)	0.82
C14:2 carnitine	0.940	(0.783, 1.129)	0.82
Bilirubin	1.040	(0.886, 1.219)	0.82
Indole-3-carboxylic acid	0.951	(0.784, 1.154)	0.82
C38:4 PE	0.952	(0.790, 1.147)	0.82
Phosphocholine	0.952	(0.787, 1.152)	0.82
Malic acid	0.957	(0.799, 1.146)	0.82

ESM Table 6 (continued) Odds ratios (OR) and 95% confidence intervals (CI) reflecting associations of circulating metabolites at baseline with retinopathy at ~15 y of follow-up in the Diabetes Prevention Program Outcomes Study (DPPOS).

Metabolite	OR[a]	95% CI	Adj. P-value[b]
C5 carnitine	1.050	(0.883, 1.248)	0.82
Anthranilic acid	1.032	(0.900, 1.183)	0.83
C7 carnitine	0.961	(0.799, 1.156)	0.84
Aspartate	0.969	(0.807, 1.163)	0.89
Kynurenic acid	0.970	(0.810, 1.161)	0.89
Betaine	0.970	(0.797, 1.180)	0.90
Sphingosine	1.015	(0.877, 1.174)	0.93
C18:0 CE	0.983	(0.825, 1.170)	0.93
Glutathione	1.018	(0.875, 1.185)	0.93
C24:1 Ceramide D18:1	1.016	(0.849, 1.216)	0.93
C22:4 CE	1.017	(0.845, 1.224)	0.93
Histidine	1.020	(0.857, 1.215)	0.93
C36:1 PC plasmalogen	0.992	(0.837, 1.176)	0.98
Hydroxyphenylpyruvic acid	0.995	(0.838, 1.181)	0.99
C22:5 CE	0.996	(0.829, 1.197)	0.99
Thiamine	1.001	(0.818, 1.226)	0.99
Methyl-hydroxyisobutyric	1.000	(0.847, 1.180)	1.00

Abbreviations: CE: cholesterol ester; DAG: Diacylglycerol; OH: hydroxyl group; PC: phosphatidylcholine; PE: phosphatidylethanolamine; PS: Phosphatidylserine; TAG: triacylglycerol

[a] From a logistic regression model weighted for the inverse probability having metabolomics data at baseline and adjusted for sex; race; time elapsed; baseline age, HbA_{1c}, BMI, and smoking status.

[b] P-values are adjusted for False Discovery Rate (FDR)

ESM Table 7 Odds ratios (OR) and 95% confidence intervals (CI) reflecting associations of circulating metabolites at baseline in relation to nephropathy at ~15 y of follow-up, stratified by original randomized treatment group, among participants in the Diabetes Prevention Program Outcomes Study (DPPOS).

Metabolite	OR[a]	95% CI	Adj. P-value[b]	Metabolite	OR[a]	95% CI	Adj. P-value[b]	Metabolite	OR[a]	95% CI	Adj. P-value[b]
Metformin				*Lifestyle*				*Placebo*			
N-carbamoyl-β-alanine	**1.987**	**(1.380, 2.991)**	0.06	C36:5 PC plasmalogen A	1.285	(1.055, 1.564)	0.23	Sarcosine	0.617	(0.433, 0.880)	0.23
C40:10 PC	0.660	(0.474, 0.920)	0.23	C22:0 SM	0.678	(0.504, 0.911)	0.23	C56:2 TAG	1.307	(1.081, 1.581)	0.23
DMGV	1.341	(1.025, 1.754)	0.24	Xanthosine	1.414	(1.054, 1.896)	0.24	C18:1 OH carnitine	1.427	(1.083, 1.880)	0.23
Citrulline	1.393	(1.036, 1.871)	0.24	3-Aminoisobutyric acid	1.348	(1.034, 1.758)	0.24	C36:0 PE	0.706	(0.513, 0.971)	0.24
Creatinine	1.509	(1.056, 2.155)	0.24	C16-OH carnitine	0.679	(0.456, 1.011)	0.27	Lactic acid	0.719	(0.540, 0.959)	0.24
C26 carnitine	1.332	(1.010, 1.757)	0.26	Arginine	0.790	(0.611, 1.023)	0.31	Xanthosine	0.677	(0.474, 0.967)	0.24
C5 DC carnitine	1.257	(1.008, 1.568)	0.26	N-carbamoyl-β-alanine	1.150	(0.979, 1.351)	0.31	C54:9 TAG	0.639	(0.411, 0.994)	0.26
C36 0 PE	1.304	(0.992, 1.713)	0.27	C54:9 TAG	1.217	(0.942, 1.572)	0.39	Malonic acid	0.669	(0.450, 0.994)	0.26
Uric acid	1.331	(0.993, 1.784)	0.27	C56:2 TAG	1.151	(0.946, 1.401)	0.40	C22:0 SM	0.755	(0.575, 0.992)	0.26
SDMA	1.315	(0.971, 1.780)	0.31	Glycine	0.813	(0.605, 1.093)	0.42	Hippuric acid	0.648	(0.404, 1.039)	0.31
Xanthosine	1.231	(0.985, 1.539)	0.31	Uric acid	0.819	(0.612, 1.095)	0.43	C9 carnitine	0.706	(0.471, 1.056)	0.31
2-Hydroxybutyric acid	0.773	(0.576, 1.037)	0.31	Lactic acid	1.174	(0.927, 1.488)	0.43	Glycine	0.758	(0.556, 1.032)	0.31
Glycine	1.247	(0.947, 1.643)	0.36	Malonic acid	1.172	(0.926, 1.485)	0.43	C14:0 SM	0.769	(0.572, 1.034)	0.31
Arginine	1.205	(0.933, 1.556)	0.40	Citrulline	1.168	(0.917, 1.487)	0.45	NMMA	0.799	(0.609, 1.048)	0.35
Keto-isocaproic acid	0.805	(0.600, 1.081)	0.40	NMMA	1.155	(0.910, 1.467)	0.49	Keto-isocaproic acid	0.783	(0.580, 1.058)	0.35
NMMA	1.182	(0.921, 1.516)	0.43	C14:0 SM	0.841	(0.622, 1.136)	0.49	Citrulline	0.764	(0.542, 1.076)	0.37
C22:1 MAG	0.863	(0.687, 1.083)	0.45	Xanthosine	0.737	(0.434, 1.251)	0.49	3-Aminoisobutyric acid	1.231	(0.936, 1.617)	0.39
ADMA	1.208	(0.894, 1.633)	0.47	C40:10 PC	1.165	(0.882, 1.540)	0.52	ADMA	0.806	(0.600, 1.081)	0.40
Hippuric acid	1.147	(0.913, 1.442)	0.49	SDMA	0.861	(0.637, 1.164)	0.56	C4 OH carnitine	1.263	(0.921, 1.731)	0.40
C22 0 SM	0.845	(0.637, 1.121)	0.49	C2 carnitine	0.889	(0.674, 1.174)	0.67	C16 OH carnitine	1.168	(0.898, 1.519)	0.49
3-Aminoisobutyric acid	1.136	(0.890, 1.450)	0.53	C22:1 MAG	0.894	(0.669, 1.194)	0.70	DMGV	0.842	(0.621, 1.143)	0.50
Xanthosine	0.822	(0.551, 1.227)	0.56	C36:0 PE	0.913	(0.704, 1.185)	0.73	N-carbamoyl-β-alanine	0.737	(0.419, 1.296)	0.52
Citrulline	1.124	(0.840, 1.504)	0.69	DMGV	1.091	(0.817, 1.457)	0.75	Arginine	0.853	(0.631, 1.153)	0.52
Sarcosine	1.100	(0.830, 1.458)	0.73	Quinolinic acid	1.098	(0.807, 1.495)	0.75	Creatinine	0.834	(0.593, 1.173)	0.52
C14:0 SM	1.104	(0.829, 1.472)	0.73	Keto-isocaproic acid	0.909	(0.676, 1.224)	0.75	C36:5 PC plasmalogen A	0.873	(0.624, 1.223)	0.69
C56:2 TAG	1.077	(0.848, 1.370)	0.75	C9 carnitine	1.065	(0.822, 1.380)	0.82	2-Hydroxybutyric acid	1.108	(0.847, 1.451)	0.70
C18:1-OH carnitine	0.900	(0.640, 1.264)	0.75	C18:1 OH carnitine	0.941	(0.682, 1.298)	0.83	C40:10 PC	0.893	(0.643, 1.241)	0.73
C16-OH carnitine	1.087	(0.821, 1.440)	0.75	2-Hydroxybutyric acid	0.949	(0.716, 1.257)	0.83	Uric acid	1.111	(0.814, 1.515)	0.73
Lactic acid	0.943	(0.707, 1.257)	0.82	ADMA	0.952	(0.708, 1.278)	0.83	SDMA	0.916	(0.680, 1.235)	0.75
Malonic acid	0.943	(0.720, 1.235)	0.82	C5 DC carnitine	0.943	(0.661, 1.346)	0.83	C26 carnitine	0.920	(0.660, 1.282)	0.81
C9 carnitine	1.050	(0.804, 1.371)	0.83	C4 OH carnitine	1.030	(0.828, 1.282)	0.86	C5 DC carnitine	0.907	(0.589, 1.399)	0.82
C2 carnitine	1.046	(0.802, 1.365)	0.83	Sarcosine	1.025	(0.809, 1.298)	0.90	Citrulline	1.062	(0.796, 1.417)	0.82
C4-OH carnitine	0.958	(0.712, 1.290)	0.86	Creatinine	1.031	(0.735, 1.446)	0.90	C22:1 MAG	1.070	(0.789, 1.450)	0.82
C36:5 PC plasmalogen A	0.980	(0.787, 1.221)	0.90	Citrulline	0.977	(0.738, 1.294)	0.90	C2 carnitine	1.056	(0.815, 1.368)	0.82
Quinolinic acid	0.982	(0.733, 1.316)	0.93	C26 carnitine	0.990	(0.749, 1.310)	0.95	Xanthosine	1.115	(0.626, 1.985)	0.83
C54:9 TAG	0.987	(0.777, 1.254)	0.93	Hippuric acid	0.997	(0.749, 1.328)	0.98	Quinolinic acid	0.972	(0.710, 1.329)	0.90

Abbreviations: ADMA: Asymmetric dimethylarginine; CE: cholesterol ester; DAG: Diacylglycerol; DMGV: Dimethylguanidino valeric acid; MAG: monoacylglycerol; NMMA: L-targinine; OH: hydroxyl group; PC: phosphatidylcholine; PE: phosphatidylethanolamine; PS: phosphatidylserine; SDMA: Symmetric dimethylarginine; TAG: triacylglycerol

[a] From a logistic regression model, weighted for the inverse probability having metabolomics data at baseline and adjusted for sex; race; time elapsed; baseline age, HbA_{1c}, BMI, and smoking status.

[b] P-values are adjusted for False Discovery Rate (FDR)

ESM Table 8 Odds ratios (OR) and 95% confidence intervals (CI) reflecting associations of circulating metabolites at baseline with neuropathy at ~15 y of follow-up, stratified by original randomized treatment group, among participants in the Diabetes Prevention Program Outcomes Study (DPPOS).

Metabolite	OR[a]	95% CI	Adj. P-value	Metabolite	OR[a]	95% CI	Adj. P-value	Metabolite	OR[a]	95% CI	Adj. P-value
Metformin				*Lifestyle*				*Placebo*			
C22:0-SM	0.538	(0.369, 0.765)	0.049*	Quinolinic acid	1.643	(1.236, 2.193)	0.049*	C56:2 TAG	1.389	(1.092, 1.768)	0.28
C36:0 PE	0.651	(0.453, 0.936)	0.28	DMGV	1.404	(1.056, 1.868)	0.28	3-Aminoisobutyric acid	0.606	(0.399, 0.920)	0.28
C14:0 SM	0.633	(0.436, 0.921)	0.28	C4 OH carnitine	1.272	(1.014, 1.596)	0.42	Citrulline	1.333	(1.066, 1.666)	0.28
2-Hydroxybutyric acid	1.338	(1.016, 1.761)	0.42	C14:0 SM	1.284	(0.969, 1.701)	0.59	C2 carnitine	1.329	(0.992, 1.779)	0.56
Citrulline	0.655	(0.419, 1.024)	0.58	C36:0 PE	1.246	(0.967, 1.605)	0.60	C26 carnitine	1.296	(0.939, 1.791)	0.63
C9 carnitine	0.716	(0.498, 1.031)	0.59	Glycine	0.766	(0.555, 1.058)	0.63	C16 OH carnitine	1.232	(0.938, 1.617)	0.65
C40:10 PC	0.706	(0.481, 1.037)	0.59	Citrulline	0.805	(0.601, 1.077)	0.65	Glycine	0.785	(0.562, 1.096)	0.67
NMMA	1.275	(0.944, 1.723)	0.63	N-carbamoyl-β-alanine	1.113	(0.958, 1.293)	0.68	DMGV	1.222	(0.909, 1.642)	0.69
N-carbamoyl-β-alanine	1.369	(0.944, 1.985)	0.63	Creatinine	0.785	(0.541, 1.139)	0.69	N-carbamoyl-β-alanine	0.680	(0.366, 1.263)	0.69
Hippuric acid	0.741	(0.505, 1.089)	0.65	Citrulline	0.826	(0.601, 1.136)	0.70	Creatinine	0.786	(0.537, 1.149)	0.69
Citrulline	0.742	(0.498, 1.106)	0.65	ADMA	0.843	(0.622, 1.144)	0.73	C22:1 MAG	0.796	(0.563, 1.126)	0.69
C26 carnitine	0.772	(0.532, 1.119)	0.69	Arginine	0.854	(0.638, 1.142)	0.74	C4 OH carnitine	1.263	(0.897, 1.778)	0.69
ADMA	1.239	(0.882, 1.740)	0.69	C26 carnitine	0.853	(0.634, 1.149)	0.74	C14:0 SM	1.188	(0.870, 1.621)	0.73
Glycine	0.791	(0.546, 1.145)	0.69	C5 DC carnitine	0.824	(0.561, 1.208)	0.77	Xanthosine	0.822	(0.568, 1.190)	0.74
Xanthosine	0.711	(0.406, 1.245)	0.70	C22:1 MAG	0.859	(0.634, 1.163)	0.77	C54:9 TAG	0.818	(0.543, 1.233)	0.77
DMGV	1.212	(0.875, 1.678)	0.70	Hippuric acid	1.123	(0.868, 1.453)	0.79	ADMA	1.167	(0.849, 1.603)	0.77
Malonic acid	0.823	(0.579, 1.169)	0.73	Sarcosine	1.110	(0.871, 1.414)	0.79	C40:10 PC	0.852	(0.592, 1.227)	0.79
Xanthosine	1.142	(0.850, 1.534)	0.79	C9 carnitine	1.121	(0.841, 1.496)	0.79	Quinolinic acid	0.865	(0.594, 1.260)	0.79
C36:5 PC plasmalogen A	0.861	(0.614, 1.208)	0.79	C40:10 PC	1.127	(0.836, 1.520)	0.79	Xanthosine	0.744	(0.356, 1.553)	0.79
C5 DC carnitine	0.876	(0.624, 1.230)	0.79	C18:1 OH carnitine	0.853	(0.596, 1.223)	0.79	C18:1 OH carnitine	1.150	(0.830, 1.594)	0.79
3-Aminoisobutyric acid	1.122	(0.845, 1.488)	0.79	C16 OH carnitine	1.122	(0.846, 1.487)	0.79	Arginine	1.099	(0.796, 1.518)	0.87
Quinolinic acid	1.085	(0.848, 1.389)	0.84	SDMA	0.900	(0.664, 1.219)	0.83	C36:0 PE	1.092	(0.799, 1.492)	0.89
C4 OH carnitine	1.105	(0.798, 1.531)	0.87	Lactic acid	1.100	(0.837, 1.447)	0.83	C5 DC carnitine	1.083	(0.728, 1.611)	0.94
Keto-isocaproic acid	1.093	(0.774, 1.543)	0.92	Keto-isocaproic acid	0.900	(0.663, 1.222)	0.83	Keto-isocaproic acid	0.939	(0.677, 1.303)	0.94
Lactic acid	0.930	(0.669, 1.293)	0.94	Xanthosine	0.860	(0.533, 1.387)	0.86	NMMA	1.038	(0.797, 1.352)	0.99
Uric acid	1.079	(0.763, 1.525)	0.94	C56:2 TAG	1.060	(0.868, 1.296)	0.87	Hippuric acid	0.972	(0.665, 1.419)	0.99
C56:2 TAG	0.935	(0.654, 1.337)	0.94	NMMA	1.062	(0.781, 1.445)	0.94	SDMA	1.032	(0.740, 1.440)	0.99
C18:1 OH carnitine	1.061	(0.771, 1.460)	0.94	Uric acid	0.930	(0.687, 1.260)	0.94	Sarcosine	1.029	(0.738, 1.434)	0.99
C16 OH carnitine	1.076	(0.787, 1.473)	0.94	3-aminoisobutyric acid	0.933	(0.674, 1.292)	0.94	C9 carnitine	1.041	(0.753, 1.440)	0.99
C54:9 TAG	0.948	(0.704, 1.277)	0.95	Xanthosine	1.061	(0.749, 1.504)	0.95	Lactic acid	1.009	(0.751, 1.355)	0.99
Arginine	0.985	(0.717, 1.354)	0.99	Malonic acid	1.041	(0.780, 1.391)	0.99	Malonic acid	0.994	(0.689, 1.432)	0.99
SDMA	0.980	(0.685, 1.404)	0.99	C54:9 TAG	0.994	(0.757, 1.307)	0.99	Citrulline	1.040	(0.735, 1.472)	0.99
Sarcosine	1.003	(0.717, 1.403)	0.99	C36:5 PC plasmalogen A	1.026	(0.768, 1.370)	0.99	Uric acid	0.984	(0.708, 1.367)	0.99
Creatinine	0.967	(0.633, 1.476)	0.99	C22:0 SM	0.970	(0.726, 1.298)	0.99	C36:5 PC plasmalogen A	0.962	(0.685, 1.352)	0.99
C22:1 MAG	1.007	(0.768, 1.322)	0.99	C2 carnitine	0.988	(0.727, 1.343)	0.99	C22:0 SM	0.998	(0.734, 1.358)	0.99
C2 carnitine	0.994	(0.722, 1.367)	0.99	2-Hydroxybutyric acid	1.032	(0.756, 1.408)	0.99	2-Hydroxybutyric acid	0.983	(0.712, 1.358)	0.99

Abbreviations: ADMA: Asymmetric dimethylarginine; CE: cholesterol ester; DAG: Diacylglycerol; DMGV: Dimethylguanidino valeric acid; MAG: monoacylglycerol; NMMA: L-targinine; OH: hydroxyl group; PC: phosphatidylcholine; PE: phosphatidylethanolamine; PS: phosphatidylserine; SDMA: Symmetric dimethylarginine; TAG: triacylglycerol

[a] From a logistic regression model, weighted for the inverse probability having metabolomics data at baseline and adjusted for sex; race; time elapsed; baseline age, HbA_{1c}, BMI, and smoking status.

* Indicates P <0.05 after False Discovery Rate (FDR) correction

ESM Table 9 Odds ratios (OR) and 95% confidence intervals (CI) reflecting associations of circulating metabolites at baseline with retinopathy at ~15 y of follow-up, stratified by original randomized treatment group, among participants in the Diabetes Prevention Program Outcomes Study (DPPOS).

Metabolite	OR[a]	95% CI	Adj. P-value[b]	Metabolite	OR[a]	95% CI	Adj. P-value[b]	Metabolite	OR[a]	95% CI	Adj. P-value[b]
Metformin				***Lifestyle***				***Placebo***			
C26 carnitine	1.488	(1.100, 2.014)	0.18	C40:10 PC	1.476	(1.109, 1.964)	0.17	C22:1 MAG	0.129	(1.271, 2.616)	0.13
2-Hydroxybutyric acid	0.622	(0.432, 0.897)	0.18	C36:0 PE	1.311	(1.003, 1.715)	0.42	Xanthosine	0.147	(1.337, 3.940)	0.15
Citrulline	1.409	(0.987, 2.012)	0.42	C26 carnitine	1.320	(0.983, 1.772)	0.42	C4 OH carnitine	0.150	(0.210, 0.762)	0.15
ADMA	1.397	(0.982, 1.986)	0.42	C54:9 TAG	1.283	(0.969, 1.698)	0.47	C18:1 OH carnitine	0.150	(0.161, 0.716)	0.15
3-Aminoisobutyric acid	1.325	(1.008, 1.740)	0.42	C9 carnitine	0.713	(0.487, 1.042)	0.47	C2 carnitine	0.236	(0.424, 0.920)	0.24
Citrulline	1.373	(0.981, 1.921)	0.42	Creatinine	0.732	(0.500, 1.071)	0.49	NMMA	0.417	(1.007, 1.647)	0.42
C18:1 OH carnitine	0.516	(0.271, 0.984)	0.42	Citrulline	0.707	(0.468, 1.068)	0.49	C9 carnitine	0.491	(0.416, 1.066)	0.49
Keto-isocaproic acid	0.720	(0.511, 1.015)	0.42	Arginine	0.789	(0.586, 1.061)	0.50	C26 carnitine	0.620	(0.897, 1.886)	0.62
Xanthosine	1.114	(0.977, 1.270)	0.49	Xanthosine	0.732	(0.494, 1.084)	0.50	Creatinine	0.620	(0.856, 1.886)	0.62
C16 OH carnitine	0.660	(0.405, 1.077)	0.49	NMMA	0.810	(0.592, 1.110)	0.62	Uric acid	0.620	(0.571, 1.112)	0.62
Lactic acid	0.773	(0.551, 1.085)	0.55	Sarcosine	0.826	(0.618, 1.103)	0.62	3-Aminoisobutyric acid	0.620	(0.525, 1.165)	0.62
N-carbamoyl-β-alanine	0.625	(0.317, 1.233)	0.62	C22:1 MAG	0.820	(0.598, 1.124)	0.62	C16 OH carnitine	0.620	(0.447, 1.178)	0.62
Quinolinic acid	0.792	(0.538, 1.164)	0.62	2-Hydroxybutyric acid	1.193	(0.893, 1.594)	0.62	Keto-isocaproic acid	0.620	(0.898, 1.843)	0.62
C22:0 SM	0.816	(0.592, 1.125)	0.62	Quinolinic acid	1.207	(0.866, 1.682)	0.63	SDMA	0.629	(0.550, 1.169)	0.63
C56:2 TAG	0.687	(0.383, 1.232)	0.62	C2 carnitine	1.182	(0.880, 1.586)	0.63	Glycine	0.629	(0.876, 1.635)	0.63
C54:9 TAG	1.167	(0.889, 1.532)	0.63	C4 OH carnitine	1.102	(0.916, 1.327)	0.64	C36:5 PC plasmalogen A	0.642	(0.861, 1.668)	0.64
C36:5 PC plasmalogen A	0.834	(0.602, 1.156)	0.64	3-Aminoisobutyric acid	1.133	(0.864, 1.484)	0.69	C36:0 PE	0.644	(0.848, 1.697)	0.64
C4 OH carnitine	0.789	(0.505, 1.232)	0.64	Malonic acid	0.873	(0.632, 1.205)	0.71	C40:10 PC	0.686	(0.824, 1.685)	0.69
C40:10 PC	0.832	(0.580, 1.194)	0.66	C36:5 PC plasmalogen A	1.097	(0.875, 1.376)	0.71	Xanthosine	0.686	(0.873, 1.431)	0.69
NMMA	1.164	(0.855, 1.584)	0.67	Citrulline	0.880	(0.627, 1.235)	0.72	C14:0 SM	0.686	(0.597, 1.218)	0.69
Creatinine	1.225	(0.811, 1.848)	0.67	N-carbamoyl-β-alanine	0.876	(0.607, 1.263)	0.72	2-Hydroxybutyric acid	0.686	(0.846, 1.599)	0.69
Hippuric acid	1.123	(0.868, 1.453)	0.69	C5 DC carnitine	1.144	(0.790, 1.656)	0.72	Lactic acid	0.712	(0.649, 1.202)	0.71
Glycine	0.858	(0.602, 1.224)	0.71	C56:2 TAG	1.091	(0.874, 1.364)	0.72	C5 DC carnitine	0.715	(0.469, 1.400)	0.72
C36:0 PE	1.133	(0.840, 1.528)	0.71	SDMA	0.904	(0.652, 1.254)	0.74	Sarcosine	0.737	(0.611, 1.290)	0.74
Arginine	0.890	(0.649, 1.218)	0.72	C18:1 OH carnitine	1.092	(0.827, 1.443)	0.74	ADMA	0.737	(0.643, 1.261)	0.74
C14:0 SM	0.886	(0.633, 1.242)	0.72	Lactic acid	1.072	(0.837, 1.373)	0.77	Hippuric acid	0.819	(0.574, 1.447)	0.82
C2 carnitine	0.884	(0.636, 1.227)	0.72	Hippuric acid	0.915	(0.641, 1.305)	0.78	C54:9 TAG	0.819	(0.732, 1.595)	0.82
SDMA	1.132	(0.793, 1.618)	0.72	Glycine	1.076	(0.810, 1.429)	0.78	Malonic acid	0.819	(0.727, 1.648)	0.82
C9 carnitine	0.895	(0.646, 1.240)	0.73	Xanthosine	0.850	(0.453, 1.594)	0.78	N-carbamoyl-β-alanine	0.819	(0.752, 1.539)	0.82
Malonic acid	0.906	(0.657, 1.249)	0.74	C16 OH carnitine	1.049	(0.867, 1.270)	0.78	Citrulline	0.819	(0.630, 1.349)	0.82
Xanthosine	0.917	(0.676, 1.244)	0.77	Uric acid	0.939	(0.693, 1.272)	0.82	C22:0 SM	0.842	(0.685, 1.305)	0.84
C5 DC carnitine	1.033	(0.857, 1.245)	0.84	C14:0 SM	1.040	(0.774, 1.398)	0.88	Arginine	0.881	(0.680, 1.351)	0.88
Sarcosine	1.044	(0.740, 1.473)	0.88	DMGV	0.972	(0.717, 1.318)	0.92		0.881	(0.758, 1.441)	0.88
C22:1 MAG	1.034	(0.778, 1.375)	0.88	C22:0 SM	0.978	(0.738, 1.296)	0.92	C56:2 TAG	0.881	(0.792, 1.355)	0.88
DMGV	1.019	(0.725, 1.431)	0.95	Keto-isocaproic acid	0.976	(0.714, 1.332)	0.92	Citrulline	0.958	(0.657, 1.473)	0.96
Uric acid	1.013	(0.725, 1.416)	0.96	ADMA	1.000	(0.731, 1.369)	1.00	Quinolinic acid	0.980	(0.687, 1.476)	0.98

Abbreviations**:** ADMA: Asymmetric dimethylarginine; CE: cholesterol ester; DAG: Diacylglycerol; DMGV: Dimethylguanidino valeric acid; MAG: monoacylglycerol; NMMA: L-targinine; OH: hydroxyl group; PC: phosphatidylcholine; PE: phosphatidylethanolamine; PS: phosphatidylserine; SDMA: Symmetric dimethylarginine; TAG: triacylglycerol

[a] From a logistic regression model, weighted for the inverse probability having metabolomics data at baseline and adjusted for sex; race; time elapsed; baseline age, HbA_{1c}, BMI, and smoking status.

[b] P-values are adjusted for False Discovery Rate (FDR)

ESM Table 10 Intersection between incident diabetes and microvascular complications among 1,947 participants in the Diabetes Prevention Program Outcome Study (DPPOS).

	Nephropathy		
	No (n = 1620)	*Yes (n = 277)*	*Missing (n = 50)*
No diabetes	672 (41.5%)	75 (27.1%)	37 (1.9%)
Diabetes	948 (58.5%)	202 (72.9%)	13 (0.7%)
	Neuropathy		
	No (n = 1377)	*Yes (n = 212)*	*Missing (n = 358)*
No diabetes	493 (35.8%)	89 (42.0%)	202 (10.4%)
Diabetes	884 (64.2%)	123 (58.0%)	156 (8.0%)
	Retinopathy		
	No (n = 1542)	*Yes (n = 194)*	*Missing (n = 211)*
No diabetes	589 (38.2%)	57 (29.4%)	138 (7.1%)
Diabetes	953 (61.8%)	137 (70.6%)	73 (3.7%)

ESM Table 11 Metabolite "hits" at baseline (*n* = 109 compounds) that predict neuropathy, nephropathy, or retinopathy among 1,840 participants in the Diabetes Prevention Program Outcomes Study (DPPOS), after excluding 107 participants with ≥1 complication at ~15 y of follow-up in the Diabetes Prevention Program Outcomes Study (DPPOS).[a]

Neuropathy	***Nephropathy***	***Retinopathy***
DMGV	2-Aminoadipic acid	1,5-Anhydroglucitol
2-Aminoadipic acid	2-Hydroxybutyric acid	2-Ketoisovaleric acid
2-Hydroxybutyric acid	Citrulline	3-Hydroxybutyric acid
3-Hydroxybutyric acid	Homogentisic acid	Adenosine diphosphate
Adenosine diphosphate	Hydroxyphenylpyruvic acid	Bilirubin
Adenosine monophosphate	Indoxyl sulfate	Deoxycholic acid
Anthranilic acid	Malic acid	Gluconic acid
Bilirubin	Orotic acid	Glycochenodeoxycholic acid
Gluconic acid	Oxalic acid	Glycocholic acid
Glucose, fructose, or galactose	Glucose or galactose	Hippuric acid
Ketoisocaproic acid	Uric acid	Indole-3-carboxylic acid
Quinolinic acid	Xanthosine	Indole-3-lactic acid
Taurocholic acid	C24:0 PC	Indoxyl sulfate
UDP GlcNAc	C34:1-PC plasmalogen B	Inosine
C40:7-PC plasmalogen	C32:0-PE	Orotic acid
C32:0 PE	C42:11-PE plasmalogen	Oxaloacetic acid
C38:2 PE	C38:4 PS	Pyruvic acid
C38:7 PE plasmalogen	C24:1 Ceramide	Methylmalonic acid
C42:11-PE plasmalogen	C18:3 CE	Uridine
C34:1 PI	C32:2 DAG	Xanthine
C38:4 PI	Serine	C34:5-PC plasmalogen
C16:1 SM	Methionine	C34:1-PC plasmalogen B
C18:0 CE	Histidine	C36:4-PC plasmalogen
C42:0 TAG	Tryptophan	C38:3-PE plasmalogen
C60:12 TAG	Dimethylglycine	C34:1 PI
Glycine	Thyroxine	C38:4 PS
Serine	Cotinine	C18:0 CE
Citrulline	5-Aminolevulinic acid	C16:1 MAG
Acetylglycine	C5-DC carnitine	C36:0 DAG
ADMA	C18:1-OH carnitine	C38:4 DAG
3-Aminoisobutyric acid	Methyl hydroxyisobutyric acid	Phenylalanine D8
1-Methylhistamine	Methylthioadenosine	Glycine
Creatinine		Serine
Adenosine		Threonine
Xanthosine		Asparagine
1-Methylnicotinamide		Arginine
Methionine sulfoxide		Tryptophan
Sarcosine		Proline
Beta-alanine		ADMA
C5 carnitine		NMMA
C7 carnitine		1-Methylhistamine
C14:2 carnitine		Niacinamide
C16-OH carnitine		Phosphocholine
C18:1-OH carnitine		Thyroxine
C20 carnitine		Cytosine
Methylthioadenosine		Xanthosine
		Pipecolic acid
		Sarcosine
		C3 carnitine
		C3-DC-CH3 carnitine
		C4 carnitine
		C4-OH carnitine
		C9 carnitine
		C10:2 carnitine
		C14 carnitine
		C16-OH carnitine
		C18:1-OH carnitine
		C26 carnitine
		Methylthioadenosine

Abbreviations: ADMA: Asymmetric dimethylarginine; CE: cholesterol ester; DAG: Diacylglycerol; DMGV: Dimethylguanidino valeric acid; MAG: monoacylglycerol; NMMA: L-targinine; OH: hydroxyl group; PC: phosphatidylcholine; PE: phosphatidylethanolamine; PS: phosphatidylserine; SDMA: Symmetric dimethylarginine; TAG: triacylglycerol

[a] Selected from bootstrapped least absolute shrinkage and selection operator (BOLASSO) model adjusted for sex; race; time elapsed during follow-up; and baseline age, HbA_{1c}, BMI, and smoking status.

ESM Table 12 Associations (odds ratios [OR] and 95% confidence intervals [CI]) of baseline metabolites predictive of neuropathy stratified by treatment arm, excluding participants (n=107) with ≥1 complication at ~15 y follow-up, in the Diabetes Prevention Program Outcomes Study (DPPOS).

Compound name	Neuropathy OR (95% CI)[a]	Adj *P*-value[b]
	Metformin	
C9 carnitine		
Model 1	0.36 (0.20, 0.65)	0.036
Model 2	0.37 (0.20, 0.61)	
Model 3	0.36 (0.19, 0.62)	
Model 4	0.36 (0.19, 0.62)	
	Lifestyle	
Serine		
Model 1	0.44 (0.28, 0.70)	0.036
Model 2	0.44 (0.27, 0.69)	
Model 3	0.44 (0.27, 0.69)	
Model 4	0.44 (0.27, 0.69)	
	Placebo	
--	--	--

[a] From a logistic regression model, weighted for the inverse probability having metabolomics data at baseline and adjusted for sex; race; time elapsed; baseline age, HbA_{1c}, BMI, and smoking status.
[b] P-values are adjusted for False Discovery Rate (FDR)
Model 1: adjusted for sex; race; time elapsed; baseline age, HbA_{1c}, BMI, and smoking status; and Diabetes Prevention Program treatment arm.
Model 2: Adjusted for Model 1 covariates + average HbA_{1c} across follow-up.
Model 3: Adjusted for Model 1 covariates + incident diabetes.
Model 4: Adjusted for Model 1 covariates + eGFR at baseline

ESM Table 13 Metabolite "hits" at baseline (n = 89 compounds) that predict each microvascular complication at ~15 y of follow-up among 952 participants who developed diabetes in the Diabetes Prevention Program Outcomes Study (DPPOS).[a]

Neuropathy	***Nephropathy***	***Retinopathy***
DMGV	3-Aminoisobutyric acid	3-Aminoisobutyric acid
1,5-Anhydroglucitol	Citrulline	Anthranilic acid
2-Hydroxyglutaric acid	Glutathione	Bilirubin
Adenosine monophosphate	Homogentisic acid	Citrulline
Anthranilic acid	Hydroxyphenylpyruvic acid	Deoxycholic acid
Citrulline	Indoxyl sulfate	D-Gluconic acid
Cyclic AMP	Ketoisocaproic acid	Fumaric acid
Fumaric acid	Orotic acid	Glutathione
Glutathione	Oxalic acid	Glycerol 3-phosphate
Homogentisic acid	Quinolinic acid	Homogentisic acid
Ketoisocaproic acid	C18:0 PE	Orotic acid
Quinolinic acid	C36:0 PC	Pantothenic acid
Glucose or galactose	C34:1 PC plasmalogen B	Succinic acid
Uridine	C36:1 PC plasmalogen	C36:0 PE
C38:2 PC	C24:1 Ceramide D18:1	C36:1 PE plasmalogen
C38:4 PS	C22:0 SM	C38:3 PE plasmalogen
C22:0 Ceramide D18:1	C18:3 CE	C34:1 PI
C18:1-CE	C22:4 CE	C38:4 PI
C18:0-CE	C32:2 DAG	C36:2 PS plasmalogen
C56:1 TAG	Serine	C36:0 DAG
Phenylalanine	Histidine	C42:0 TAG
Glycine	Phenylalanine	Asparagine
Serine	Hydroxyproline	Histidine
Glutamine	Ornithine	Arginine
ADMA	N-Methylmalonamic acid	Symmetric dimethylarginine
Phosphocholine	1-Methylhistamine	N-Methylmalonamic acid
Creatine	N-Carbamoyl beta alanine	Creatine
Adenosine	Niacinamide	Thyroxine
1-Methylnicotinamide	Choline	Cytosine
Methionine sulfoxide	Phosphocholine	Xanthosine
Sarcosine	Creatine	Pipecolic acid
Beta alanine	Adenosine	5-Aminolevulinic acid
C16 carnitine	Cotinine	Sarcosine
Methylthioadenosine	Butyrobetaine	C3 carnitine
	C5-DC carnitine	C4-OH carnitine
	C16-OH carnitine	C9 carnitine
	C18 carnitine	C26 carnitine
	Methyl hydroxyisobutyric	
	Methylthioadenosine	

Abbreviations: ADMA: Asymmetric dimethylarginine; CE: cholesterol ester; DAG: Diacylglycerol; DMGV: Dimethylguanidino valeric acid; MAG: monoacylglycerol; NMMA: L-targinine; OH: hydroxyl group; PC: phosphatidylcholine; PE: phosphatidylethanolamine; PS: phosphatidylserine; SDMA: Symmetric dimethylarginine; TAG: triacylglycerol

a Selected from bootstrapped least absolute shrinkage and selection operator (LASSO) model adjusted for sex; race; time elapsed during follow-up; and baseline age, HbA_{1c}, BMI, and smoking status.

ESM Table 14 Odds ratios (OR) and 95% confidence intervals (CI) reflecting associations of circulating metabolites at baseline with prevalent microvascular complications at ~15-y of follow-up among 952 participants who developed diabetes in the Diabetes Prevention Program Outcomes Study (DPPOS).

Compound name	OR (95% CI)[a]	Adj *P*-value[a]
	Nephropathy	
C22:0 SM		
Model 1	0.65 (0.53, 0.79)	0.001*
Model 2	0.64 (0.53, 0.78)	
Model 3	0.72 (0.60, 0.85)	
Serine		
Model 1	0.71 (0.59, 0.87)	0.030*
Model 2	0.72 (0.59, 0.87)	
Model 3	0.71 (0.58, 0.87)	
1-Methylhistamine		
Model 1	1.29 (1.10, 1.52)	0.031*
Model 2	1.29 (1.09, 1.52)	
Model 3	1.29 (1.10, 1.52)	
Histidine		
Model 1	0.74 (0.61, 0.89)	0.031*
Model 2	0.73 (0.60, 0.88)	
Model 3	0.73 (0.60, 0.88)	
C56:1 TAG		
Model 1	1.25 (1.08, 1.44)	0.033*
Model 2	1.25 (1.08, 1.45)	
Model 3	1.24 (1.07, 1.43)	
C36:1 PC plasmalogen		
Model 1	0.75 (0.63, 0.91)	0.033*
Model 2	0.76 (0.63, 0.91)	
Model 3	0.76 (0.63, 0.91)	
	Retinopathy	
C26 carnitine		
Model 1	1.44 (1.17, 1.76)	0.046*
Model 2	1.44 (1.16, 1.77)	
Model 3	1.40 (1.13, 1.71)	

[a]P-values are presented only for Model 1 as this model was used to identify metabolites associated with outcomes after False Discovery Rate (FDR) correction.

* Indicates P <0.05 after FDR correction.

Model 1: Adjusted for sex; race; time elapsed; baseline age, HbA_{1c}, BMI, and smoking status; and Diabetes Prevention Project treatment arm.

Model 2: Model 1 covariates + average HbA_{1c} across follow-up.

Model 3: Model 1 covariates + baseline estimated glomerular filtration rate (eGFR)

ESM Figure 1 Distribution of 109 metabolites associated with microvascular complications among 1,840 participants in the Diabetes Prevention Program Outcomes Study (DPPOS), excluding participants (n=107) with ≥1 complication at ~15 y of follow-up, in the Diabetes Prevention Program Outcomes Study (DPPOS).

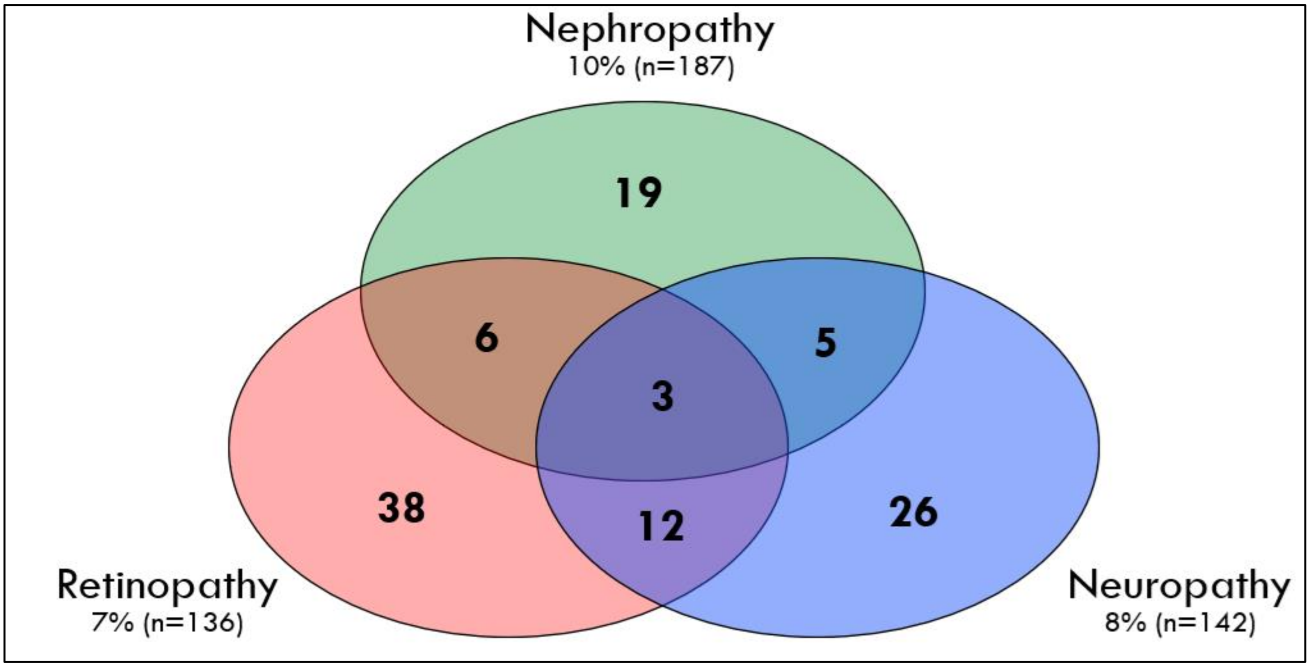

DPP, DPPOS I & DPPOS II Research Group
(1996-2013)

Pennington Biomedical Research Center (Baton Rouge, LA)
George A. Bray, MD*
Kishore M. Gadde, MD*
Iris W. Culbert, BSN, RN, CCRC**
Jennifer Arceneaux RN, BSN**
Annie Chatellier, RN, CCRC**
Amber Dragg RD, LDN**
Catherine M. Champagne, PhD, RD
Crystal Duncan, LPN
Barbara Eberhardt, RD, LDN
Frank Greenway, MD
Fonda G. Guillory, LPN
April A. Herbert, RD
Michael L. Jeffirs, LPN
Betty M. Kennedy, MPA
Erma Levy, RD
Monica Lockett, LPN
Jennifer C. Lovejoy, PhD
Laura H. Morris, BS
Lee E. Melancon, BA, BS
Donna H. Ryan, MD
Deborah A. Sanford, LPN
Kenneth G. Smith, BS, MT
Lisa L. Smith, BS
Julia A. St.Amant, RTR
Richard T. Tulley, PhD
Paula C. Vicknair, MS, RD
Donald Williamson, PhD
Jeffery J. Zachwieja, PhD

University of Chicago (Chicago, IL)
Kenneth S. Polonsky, MD*
Janet Tobian, MD, PhD*
David A. Ehrmann, MD*
Margaret J. Matulik, RN, BSN**
Bart Clark, MD
Kirsten Czech, MS
Catherine DeSandre, BA
Ruthanne Hilbrich, RD
Wylie McNabb, EdD
Ann R. Semenske, MS, RD

Jefferson Medical College (Philadelphia, PA)
Jose F. Caro, MD*
Kevin Furlong, DO*
Barry J. Goldstein, MD, PhD*
Pamela G. Watson, RN, ScD*
Kellie A. Smith, RN, MSN**
Jewel Mendoza, RN, BSN**
Wendi Wildman, RN**
Renee Liberoni, MPH
John Spandorfer, MD
Constance Pepe, MS, RD

University of Miami (Miami, FL)
Richard P. Donahue, PhD*
Ronald B. Goldberg, MD*
Ronald Prineas, MD, PhD*
Jeanette Calles, MSEd**
Juliet Ojito, RN**
Patricia Rowe, MPA**
Paul Cassanova-Romero, MD
Sumaya Castillo-Florez, MPH
Hermes J. Florez, MD
Anna Giannella, RD, MS**
Lascelles Kirby, MS
Carmen Larreal
Olga Lara
Valerie McLymont, RN
Jadell Mendez
Arlette Perry, PhD
Patrice Saab, PhD
Beth Veciana

The University of Texas Health Science Center (San Antonio, TX)
Steven M. Haffner, MD, MPH*
Helen P. Hazuda, PhD*
Maria G. Montez, RN, MSHP, CDE**
Kathy Hattaway, RD, MS
Carlos Lorenzo, MD, PhD
Arlene Martinez, RN, BSN, CDE
Tatiana Walker, RD, MS, CDE

University of Colorado (Denver, CO)
Dana Dabelea, MD, PhD*
Richard F. Hamman, MD, DrPH*
Patricia V. Nash, MS**
Sheila C. Steinke, MS**
Lisa Testaverde, MS**
Denise R. Anderson, RN, BSN
Larry B. Ballonoff, MD
Alexis Bouffard, MA, RN, BSN
Brian Bucca OD, FAOO
B. Ned Calonge, MD, MPH
Lynne Delve
Martha Farago, RN
James O. Hill, PhD
Shelley R. Hoyer, BS
Tonya Jenkins, RD, CDE
Bonnie T. Jortberg, MS, RD, CDE
Dione Lenz, RN, BSN, CDE
Marsha Miller, MS, RD
Leigh Perreault, MD
David W. Price, MD
Judith G. Regensteiner, PhD

* denotes Principal Investigator
** denotes Program Coordinator

Helen Seagle, MS, RD
Carissa M. Smith, BS
Brent VanDorsten, PhD
Joslin Diabetes Center (Boston, MA)
Edward S. Horton, MD*
Kathleen E. Lawton, RN**
Catherine S. Poirier, RN, BSN**
Kati Swift, RN, BSN**
Ronald A. Arky, MD
Marybeth Bryant
Jacqueline P. Burke, BSN
Enrique Caballero, MD
Karen M. Callaphan, BA
Barbara Fargnoli, RD
Therese Franklin
Om P. Ganda, MD
Ashley Guidi, BS
Mathew Guido, BA
Sharon D. Jackson, MS, RD, CDE
Alan M. Jacobsen, MD
Lori Lambert, MS, RD, LD
Sarah Ledbury, Med, RD
Margaret Kocal, RN, CDE
Lyn M. Kula, RD
Maureen A. Malloy, BS
Maryanne Nicosia, MS, RD
Cathryn F. Oldmixon, RN
Jocelyn Pan, BS, MPH
Marizel Quitingon
Stacy Rubtchinsky, BS
Jessica Sansoucy, BS
Dana Schweizer, BSN
Ellen W. Seely, MD
Donald Simonson, MD
Fannie Smith, MD
Caren G. Solomon, MD, MPH
Jeanne Spellman, RD
James Warram, MD
VA Puget Sound Health Care System and University of Washington (Seattle, WA)
Steven E. Kahn, MB, ChB*
Brenda K. Montgomery, RN, BSN, CDE**
Wilfred Fujimoto, MD
Robert H. Knopp, MD
Edward W. Lipkin, MD
Michelle Marr, BA
Ivy Morgan-Taggart
Anne Murillo, BS
Dace Trence, MD
Lonnese Taylor, RN, BS
April Thomas, RD, MPH, CDE
Elaine C. Tsai, MD, MPH
University of Tennessee (Memphis, TN)
Samuel Dagogo-Jack, MD, MSc, FRCP, FACP*
Abbas E. Kitabchi, PhD, MD, FACP*
Mary E. Murphy, RN, MS, CDE, MBA**
Laura Taylor, RN, BSN, CDE**
Jennifer Dolgoff, RN, BSN**
William B. Applegate, MD, MPH
Michael Bryer-Ash, MD
Debra Clark, LPN
Sandra L. Frieson, RN
Uzoma Ibebuogu, MD
Raed Imseis, MD
Helen Lambeth, RN, BSN
Lynne C. Lichtermann, RN, BSN
Hooman Oktaei, MD
Harriet Ricks
Lily M.K. Rutledge, RN, BSN
Amy R. Sherman, RD, LD
Clara M. Smith, RD, MHP, LDN
Judith E. Soberman, MD
Beverly Williams-Cleaves, MD
Northwestern University's Feinberg School of Medicine (Chicago, IL)
Boyd E. Metzger, MD*
Mark E. Molitch, MD*
Mariana K. Johnson, MS, RN**
Daphne T. Adelman, MBA, RN
Catherine Behrends
Michelle Cook, MS
Marian Fitzgibbon, PhD
Mimi M. Giles, MS, RD
Deloris Heard, MA
Cheryl K.H. Johnson, MS, RN
Diane Larsen, BS
Anne Lowe, BS
Megan Lyman, BS
David McPherson, MD
Samsam C. Penn, BA
Thomas Pitts, MD
Renee Reinhart, RN, MS
Susan Roston, RN, RD
Pamela A. Schinleber, RN, MS
Massachusetts General Hospital (Boston, MA)
David M. Nathan, MD*
Charles McKitrick, BSN**
Heather Turgeon, BSN**
Mary Larkin, MSN**
Kathy Abbott
Ellen Anderson, MS, RD
Laurie Bissett, MS, RD

* denotes Principal Investigator
** denotes Program Coordinator

DPP, DPPOS I & DPPOS II Research Group
(1996-2013)

Kristy Bondi, BS
Enrico Cagliero, MD
Jose C. Florez, MD, PhD+
Kali D'Anna
Linda Delahanty, MS, RD
Valerie Goldman, MS, RD
Peter Lou, MD
Alexandra Poulos
Elyse Raymond, BS
Christine Stevens, RN
Beverly Tseng

University of California-San Diego (La Jolla, CA)

Jerrold M. Olefsky, MD*
Elizabeth Barrett-Connor, MD*
Mary Lou Carrion-Petersen, RN, BSN**
Madeline Beltran, RN, BSN, CDE
Lauren N. Claravall, BS
Jonalle M. Dowden, BS
Steven V. Edelman, MD
Robert R. Henry, MD
Javiva Horne, RD
Marycie Lamkin, RN
Simona Szerdi Janesch, BA
Diana Leos
Sunder Mudaliar, MD
William Polonsky, PhD
Jean Smith, RN
Jennifer Torio-Hurley
Karen Vejvoda, RN, BSN, CDE, CCRC

Columbia University (New York, NY)

F. Xavier Pi-Sunyer, MD*
Jane E. Lee, MS**
David B. Allison, PhD
Nnenna Agharanya
Nancy J. Aronoff, MS, RD
Maria Baldo
Jill P. Crandall, MD
Sandra T. Foo, MD
Susan Hagamen, MS, RN, CDE
Jose A. Luchsinger, MD, MPH
Carmen Pal, MD
Kathy Parkes, RN
Mary Beth Pena, RN
Ellen S. Rooney, BA
Gretchen E.H. Van Wye, MA
Kristine A. Viscovich, ANP

Indiana University (Indianapolis, IN)

David G. Marrero, PhD*
Kieren J. Mather, MD*
Melvin J. Prince, MD*
Susie M. Kelly, RN, CDE**
Marcia A. Jackson**
Gina McAtee**
Paula Putenney, RN**
Ronald T. Ackermann, MD
Carolyn M. Cantrell
Yolanda F. Dotson, BS
Edwin S. Fineberg, MD
Megan Fultz
John C. Guare, PhD
Angela Hadden
James M. Ignaut, MA
Marion S. Kirkman, MD
Erin O'Kelly Phillips
Beverly D. Porter, MSN
Paris J. Roach, MD
Nancy D. Rowland, BS, MS
Madelyn L. Wheeler, RD

Medstar Research Institute (Washington, DC)

Vanita Aroda, MD*
Robert E. Ratner, MD*
Gretchen Youssef, RD, CDE**
Sue Shapiro, RN, BSN, CCRC**
Catherine Bavido-Arrage, MS, RD, LD
Geraldine Boggs, MSN, RN
Marjorie Bronsord, MS, RD, CDE
Ernestine Brown
Wayman W. Cheatham, MD
Susan Cola
Cindy Evans
Peggy Gibbs
Tracy Kellum, MS, RD, CDE
Renee Wiggins, RD
Milvia Lagarda
Lilia Leon
Claresa Levatan, MD
Milajurine Lindsay
Asha K. Nair, BS
Maureen Passaro, MD
Angela Silverman
Gabriel Uwaifo, MD
Debra Wells-Thayer, NP, CDE

University of Southern California/UCLA Research Center (Alhambra, CA)

Mohammed F. Saad, MD*
Karol Watson, MD*
Maria Budget**
Sujata Jinagouda, MD**
Medhat Botrous, MD**
Khan Akbar, MD
Claudia Conzues
Perpetua Magpuri

* denotes Principal Investigator
** denotes Program Coordinator

DPP, DPPOS I & DPPOS II Research Group
(1996-2013)

Kathy Ngo
Amer Rassam, MD
Debra Waters
Kathy Xapthalamous
Washington University (St. Louis, MO)
Julio V. Santiago, MD*
Samuel Dagogo-Jack, MD, MSc, FRCP, FACP*
Neil H. White, MD, CDE*
Angela L. Brown, MD*
Samia Das, MS, MBA, RD, LD**
Prajakta Khare-Ranade, MSc, RDN, LD**
Tamara Stich, RN, MSN, CDE**
Ana Santiago, RN
Edwin Fisher, PhD
Emma Hurt, RN
Tracy Jones, RN
Michelle Kerr, RD
Lucy Ryder, RN
Cormarie Wernimont, RD, LD
Johns Hopkins School of Medicine (Baltimore, MD)
Sherita Hill Golden, MD, MHS, FAHA*
Christopher D. Saudek, MD*
Vanessa Bradley, BA**
Emily Sullivan, MEd, RN**
Tracy Whittington, BS**
Caroline Abbas
Adrienne Allen
Frederick L. Brancati, MD, MHS
Sharon Cappelli
Jeanne M. Clark, MD
Jeanne B. Charleston, RN, MSN
Janice Freel
Katherine Horak, RD
Alicia Greene
Dawn Jiggetts
Deloris Johnson
Hope Joseph
Kimberly Loman
Henry Mosley
John Reusing
Richard R. Rubin, PhD
Alafia Samuels, MD
Thomas Shields
Shawne Stephens
Kerry J. Stewart, EdD
LeeLana Thomas
Evonne Utsey
Paula Williamson
University of New Mexico (Albuquerque, NM)
David S. Schade, MD*
Karwyn S. Adams, RN, MSN**
Janene L. Canady, RN, CDE**
Carolyn Johannes, RN, CDE**
Claire Hemphill, RN, BSN**
Penny Hyde, RN, BSN**
Leslie F. Atler, PhD
Patrick J. Boyle, MD
Mark R. Burge, MD
Lisa Chai, RN
Kathleen Colleran, MD
Ysela Gonzales
Doris A. Hernandez-McGinnis
Patricia Katz, LPN
Carolyn King, Med
Amer Rassam, MD
Sofya Rubinchik, MD
Willette Senter, RD
Debra Waters, PhD
Albert Einstein College of Medicine (Bronx, NY)
Jill Crandall, MD*
Harry Shamoon, MD*
Janet O. Brown, RN, MPH, MSN**
Gilda Trandafirescu, MD**
Elsie Adorno, BS
Liane Cox, MS, RD
Helena Duffy, MS, C-ANP
Samuel Engel, MD
Allison Friedler, BS
Angela Goldstein, FNP-C, NPP, CSW
Crystal J. Howard-Century, MA
Jennifer Lukin, BA
Stacey Kloiber, RN
Nadege Longchamp, LPN
Helen Martinez, RN, MSN, FNP-C
Dorothy Pompi, BA
Jonathan Scheindlin, MD
Elissa Violino, RD, MS
Elizabeth A. Walker PhD, RN
Judith Wylie-Rosett, EdD, RD
Elise Zimmerman, RD, MS
Joel Zonszein, MD
University of Pittsburgh (Pittsburgh, PA)
Trevor Orchard, MD*
Rena R. Wing, PhD*
Susan Jeffries, RN, MSN**
Gaye Koenning, MS, RD**
M. Kaye Kramer, BSN, MPH**
Marie Smith, RN, BSN**
Susan Barr, BS
Catherine Benchoff
Miriam Boraz, PhD

* denotes Principal Investigator
** denotes Program Coordinator

DPP, DPPOS I & DPPOS II Research Group
(1996-2013)

Lisa Clifford, BS
Rebecca Culyba, BS
Marlene Frazier
Ryan Gilligan, BS
Stephanie Guimond, BS
Susan Harrier, MLT
Louann Harris, RN
Andrea Kriska, PhD
Qurashia Manjoo, MD
Monica Mullen, MHP, RD
Alicia Noel, BS
Amy Otto, PhD
Jessica Pettigrew, CMA
Bonny Rockette-Wagner, PhD
Debra Rubinstein, MD
Linda Semler, MS, RD
Cheryl F. Smith, PhD
Elizabeth Venditti, PhD
Valarie Weinzierl, MPH
Katherine V. Williams, MD, MPH
Tara Wilson, BA

University of Hawaii (Honolulu, HI)

Richard F. Arakaki, MD*
Renee W. Latimer, BSN, MPH** Narleen K. Baker-Ladao, BS**
Mae K. Isonaga, RD, MPH**
Ralph Beddow, MD
Nina E. Bermudez, MS
Lorna Dias, AA
Jillian Inouye, RN, PhD
Marjorie K. Mau, MD
John S. Melish, MD
Kathy Mikami, BS, RD
Pharis Mohideen, MD
Sharon K. Odom, RD, MPH
Raynette U. Perry, AA
Robin E. Yamamoto, CDE, RD

Southwest American Indian Centers (Phoenix, AZ; Shiprock, NM; Zuni, NM)

William C. Knowler, MD, DrPH*+
Norman Cooeyate**
Mary A. Hoskin, RD, MS**
Carol A. Percy, RN, MS**
Alvera Enote**
Camille Natewa**
Kelly J. Acton, MD, MPH
Vickie L. Andre, RN, FNP
Rosalyn Barber
Shandiin Begay, MPH
Peter H. Bennett, MB, FRCP
Mary Beth Benson, RN, BSN
Evelyn C. Bird, RD, MPH
Brenda A. Broussard, RD, MPH, MBA, CDE
Brian C. Bucca, OD, FAAO
Marcella Chavez, RN, AS
Sherron Cook
Jeff Curtis, MD
Tara Dacawyma
Matthew S. Doughty, MD
Roberta Duncan, RD
Charlotte Dodge
Cyndy Edgerton, RD
Jacqueline M. Ghahate
Justin Glass, MD
Martia Glass, MD
Dorothy Gohdes, MD
Wendy Grant, MD
Robert L. Hanson, MD, MPH
Ellie Horse
Louise E. Ingraham, MS, RD, LN
Merry Jackson
Priscilla Jay
Roylen S. Kaskalla
David Kessler, MD
Kathleen M. Kobus, RNC-ANP
Jonathan Krakoff, MD
Jason Kurland, MD
Catherine Manus, LPN
Cherie McCabe
Sara Michaels, MD
Tina Morgan
Yolanda Nashboo
Julie A. Nelson, RD
Steven Poirier, MD
Evette Polczynski, MD
Christopher Piromalli, DO
Mike Reidy, MD
Jeanine Roumain, MD, MPH
Debra Rowse, MD
Robert J. Roy
Sandra Sangster, RD
Janet Sewenemewa
Miranda Smart
Darryl Tonemah, PhD
Rachel Williams, FNP
Charlton Wilson, MD
Michelle Yazzie

George Washington University Biostatistics Center (DPP Coordinating Center Rockville, MD)

Raymond Bain, PhD*
Sarah Fowler, PhD*

* denotes Principal Investigator
** denotes Program Coordinator

DPP, DPPOS I & DPPOS II Research Group
(1996-2013)

Marinella Temprosa, PhD*
Michael D. Larsen, PhD*
Tina Brenneman**
Sharon L. Edelstein, ScM**
Solome Abebe, MS
Julie Bamdad, MS
Melanie Barkalow
Joel Bethepu, MPH
Tsedenia Bezabeh, MS
Nicole Butler, MPH
Jackie Callaghan
Caitlin E. Carter, MPH
Costas Christophi, PhD
Gregory M. Dwyer, MPH
Mary Foulkes, PhD
Yuping Gao
Robert Gooding
Adrienne Gottlieb
Kristina L. Grimes
Nisha Grover-Fairchild, MPH
Lori Haffner, MS
Heather Hoffman, PhD
Kathleen Jablonski, PhD
Steve Jones
Tara L. Jones
Richard Katz, MD
Preethy Kolinjivadi, MS
John M. Lachin, ScD
Yong Ma, PhD
Pamela Mucik
Robert Orlosky
Qing Pan, PhD
Susan Reamer
James Rochon, PhD
Alla Sapozhnikova
Hanna Sherif, MS
Charlotte Stimpson
Ashley Hogan Tjaden, MPH
Fredricka Walker-Murray

Lifestyle Resource Core

Elizabeth M. Venditti, PhD*
Andrea M. Kriska, PhD
Linda Semler, MS, RD, LDN
Valerie Weinzierl, MPH

Central Biochemistry Laboratory (Seattle, WA)

Santica Marcovina, PhD, ScD*
Jessica Harting**
F. Alan Aldrich**
John Albers, PhD
Greg Strylewicz, PhD

NIH/NIDDK (Bethesda, MD)

R. Eastman, MD
Judith Fradkin, MD
Sanford Garfield, PhD
Christine Lee, MD, MS

Centers for Disease Control & Prevention (Atlanta, GA)

Edward Gregg, PhD
Ping Zhang, PhD

Carotid Ultrasound

Dan O'Leary, MD*
Gregory Evans

Coronary Artery Calcification Reading Center

Matthew Budoff, MD
Chris Dailing

CT Scan Reading Center

Elizabeth Stamm, MD*

Dual Energy X-ray Absorptiometry Reading Center (San Francisco, CA)

Ann Schwartz, PhD
Caroline Navy
Lisa Palermo, MS

Epidemiological Cardiology Research Center-Epicare (Winston-Salem, NC)

Pentti Rautaharju, MD, PhD*
Ronald J. Prineas, MD, PhD**
Teresa Alexander
Charles Campbell, MS
Sharon Hall
Yabing Li, MD
Margaret Mills
Nancy Pemberton, MS
Farida Rautaharju, PhD
Zhuming Zhang, MD
Elsayed Z. Soliman, MD*
Julie Hu, MSc
Susan Hensley, BS
Lisa Keasler
Tonya Taylor

Fundus Photo Reading Center (Madison, WI)

Ronald Danis, MD*
Matthew Davis, MD*
Larry Hubbard*
Ryan Endres**
Deborah Elsas**
Samantha Johnson**
Vonnie Gama
Anne Goulding

Neurocognitive Assessment Group

Jose A. Luchsinger, MD, MPH
Jennifer Manly, PhD

Nutrition Coding Center (Columbia, SC)

* denotes Principal Investigator
** denotes Program Coordinator

DPP, DPPOS I & DPPOS II Research Group
(1996-2013)

Elizabeth Mayer-Davis, PhD*
Robert R. Moran, PhD**

Quality of Well-Being Center (La Jolla, CA)
Ted Ganiats, MD*
Kristin David, MHP*
Andrew J. Sarkin, PhD*
Erik Groessl, PhD
Naomi Katzir

University of Michigan (Ann Arbor, MI)
William H. Herman, MD, MPH
Michael Brändle, MD, MS
Morton B. Brown, PhD

+Genetics Working Group
Jose C. Florez, MD, PhD[1, 2]
David Altshuler, MD, PhD[1, 2]
Liana K. Billings, MD[1]
Ling Chen, MS[1]
Maegan Harden, BS[2]
Robert L. Hanson, MD, MPH[3]
William C. Knowler, MD, DrPH[3]
Toni I. Pollin, PhD[4]
Alan R. Shuldiner, MD[4]
Kathleen Jablonski, PhD[5]
Paul W. Franks, PhD, MPhil, MS[6, 7, 8]
Marie-France Hivert, MD[9]

1=Massachusetts General Hospital
2=Broad Institute
3=NIDDK
4=University of Maryland
5=Coordinating Center
6=Lund University, Sweden
7=Umeå University, Sweden
8=Harvard School of Public Health
9=Université de Sherbrooke

* denotes Principal Investigator
** denotes Program Coordinator
